# Exhalatory dynamic interactions between patients connected to a shared ventilation device

**DOI:** 10.1371/journal.pone.0250672

**Published:** 2021-05-04

**Authors:** Pedro M. Garcia Eijo, Juan D’Adamo, Arturo Bianchetti, Thomas Duriez, Juan M. Cabaleiro, Célica Irrazabal, Pablo Otero, Guillermo Artana

**Affiliations:** 1 Laboratorio de Fluidodinámica, Facultad de Ingeniería, Universidad de Buenos Aires-CONICET, Buenos Aires, Argentina; 2 División Terapia Intensiva del Hospital de Clínicas, Universidad de Buenos Aires, Buenos Aires, Argentina; 3 Cátedra de Anestesiología y Algiología, Facultad de Ciencias Veterinarias, Universidad de Buenos Aires, Buenos Aires, Argentina; University of Nottingham, UNITED KINGDOM

## Abstract

In this work a shared pressure-controlled ventilation device for two patients is considered. By the use of different valves incorporated to the circuit, the device enables the restriction of possible cross contamination and the individualization of tidal volumes, driving pressures, and positive end expiratory pressure PEEP. Possible interactions in the expiratory dynamics of different pairs of patients are evaluated in terms of the characteristic exhalatory times. These characteristic times can not be easily established using simple linear lumped element models. For this purpose, a 1D model using the Hydraulic and Mechanical libraries in Matlab Simulink was developed. In this sense, experiments accompany this study to validate the model and characterize the different valves of the circuit. Our results show that connecting two patients in parallel to a ventilator always resulted in delays of time during the exhalation. The size of this effect depends on different parameters associated with the patients, the circuit and the ventilator. The dynamics of the exhalation of both patients is determined by the ratios between patients exhalatory resistances, compliances, driving pressures and PEEPs. Adverse effects on exhalations became less noticeable when respiratory parameters of both patients were similar, flow resistances of valves added to the circuit were negligible, and when the ventilator exhalatory valve resistance was also negligible. The asymmetries of driving pressures, compliances or resistances exacerbated the possibility of auto-PEEP and the increase in relaxation times became greater in one patient than in the other. In contrast, exhalatory dynamics were less sensitive to the ratio of PEEP imposed to the patients.

## Introduction

Different critical situations can locally generate a collapse in the health care system. In such circumstances, there may be a time window where hospitals could need to provide ventilatory support to a number of patients greater than the existing number of ventilators. This situation can be further aggravated by the fact that intensive care ventilators are very expensive and, in many hospitals, their surplus is limited.

As a last resort, systems based on the possibility to share one ventilator between two patients (shared ventilation or also dual ventilation), have been proposed since 2000 [1] and revisited later by different authors (see, for instance, [2, 3]). This strategy may produce a buffer in the capacity of hospitals to treat patients with respiratory diseases and therefore allow for a more flexible policy on ventilators stockpiles.

As a consequence of the COVID-19 pandemic, many countries have seen supply shortages, and hospitals were urged to find options to maximize the utilization of available ventilators. In this context, guidelines to support the implementation of shared ventilation were proposed in Europe (Protocol CHRU Brest (France) and Comision Ingenieria Médica y Sanitaria Madrid (Spain)) and the USA (Mount Sinai and NY Presbyterian Hospitals protocols). In this last country, considering the context of urgency, the US Food and Drug Administration provided Emergency Use Authorization for devices that facilitated ventilator sharing. In consonance with previous articles (see [4–6]), some American medical societies recommend against this use of ventilators because of safety, technical challenges, and ethical concerns. In contrast, during the shortages they proposed triage-based ventilator allocation to patients most likely to benefit and survive (see [7]).

The major disadvantages stated in these documents can be summarized as follows: 1) inability to adequately control ventilatory variables such as tidal Volume (*V*_*T*_) and PEEP for each individual patient, 2) absence of autonomy as a change in respiratory mechanics in one patient would adversely affect ventilation to the co-ventilated patient(s), and 3) the possibility of cross contamination between patients.

These legitimate concerns may apply to shared ventilation systems composed by circuits in which only splitters are present as those described by [8]. Some authors [9], have referred to this kind of strategy as naive sharing. At the same time, a large number of multidisciplinary groups worldwide agreed [9–12] that the objections related to technical and safety problems can be solved by incorporating adequate valves in the inspiratory and expiratory limbs of the circuit.

Considering devices with such improvements, it has been verified in tests produced with test lungs that: 1) the inspiratory pressure peak can be adjusted for each patient 2)adjustable in-line PEEP valves allow to set a differential PEEP for each patient; 3) the pressure and/or volume curve for each patient can be monitored; 4) the system retains the high-pressure alarms of the ventilator and a disconnection signal can be issued when carefully setting a volume minute ventilation alarm.

Still, some drawbacks arise from these kinds of systems: (1) Limited ventilation modes (pressure control mode is accepted as the safest mode to be used) [9], (2) patients must be paralysed; (3) parameters such as breath rate (BR) and inspiration-expiration ratio (I:E) must be shared; (4) methods for individualizing fraction of inspired oxygen (FiO_2_) have not yet been developed; (5) increased compressible volume; (6) potential increased risk of auto-PEEP (air flow at the end of exhalation); (7) off-label use of mechanical ventilators; and (8) increased complexity of the patient-ventilator-breathing circuit system.

An important characteristic in the design of dual ventilation devices is that they should operate safely with any ventilator. The ventilator feedback system relies on sensors measuring parameters (pressure and flow in general) located in the inspiratory and/or expiratory circuit. The control strategies to adapt the ventilator output to any perturbation are based on these signals. Unfortunately, these strategies are not the same for all ventilators. Much of the previous research has not carefully explained how the control system may be affected when dual ventilation devices are connected (see, for instance, [13]). Furthermore, it is not clear whether the different devices proposed are universal or ventilator type-specific.

At present, dual ventilation strategies lack a substantial amount of mandatory reliable clinical studies. The possibility of performing research on actual patients in a clinical environment is not feasible given current circumstances. In the past, *in vivo* tests were performed with healthy animals [14] or volunteers [15] that may not strictly reflect the ventilation dynamics on severe acute respiratory distress conditions. Nevertheless, these kinds of studies may provide a rationale for action during critical ventilator shortages. More recently, a group of researchers [16] reported an observational study of four patients with acute respiratory failure due to COVID-19. Tests were performed during the course of an hour with satisfactory results using a device that did not allow to control individual PEEP of patients.

This paper considers a dual ventilation device named ‘ACRA’ (acronym for Enhanced Capacity of Mechanical Ventilators, in Spanish) that works by setting the ventilator on the pressure controlled mode and provides individualized inspiratory pressures and PEEP control for two patients.

An important feature of the circuit ACRA is that special care has been dedicated to obtain a device compatible with controllers of different ventilators. One main drawback is that because of the different valves incorporated to the system, the flow resistance in the exhalatory limbs has been noted to increase. Additionally, the use of a single in line PEEP valve produces a fluid dynamic asymmetry in the exhalation limbs of the patients.

Alterations produced in the exhalation dynamics and the possibility of auto-PEEP of patients connected to a shared ventilation device require an analysis that is absent at present. Even for perfectly identical patients, any shared ventilation scheme will bring consequences in expiratory dynamics for both of them. This, is associated with the confluence of both expiratory limbs to a single node that forces the pressure to be the same at that point for all branches. Therefore, there is a coupling of circuits and individual exhalation dynamics are no longer independent. Furthermore, the exhalatory valve resistance is a parameter that may enlarge the expiration duration for both patients, as the flow traversing this restriction is largely increased when compared to individual ventilation. This effect significantly depends on, among other parameters, the ventilator considered.

The coupled dynamics of exhalation indicate that the phenomena should be determined by the values of different ratios of parameters associated with the patients and with the ventilation requirements.

Given the complexity involved, research of the fluid dynamics enlightens possibilities and limitations of dual ventilation systems. A better understanding of the underlying fluid dynamics behaviour of the ventilator-circuit-patient system can be achieved through numerical models. Furthermore, with adequate fluid dynamics numerical models, data bases associated with different clinical scenarios can be easily enlarged.

Previous research concerning computational modelling of dual ventilation systems has proposed to analyse the delivered tidal volumes. In [9], a simplified model has been used to compare dual naive ventilation and dual ventilation systems operating in volume control ventilation mode and pressure control ventilation mode. A linear lumped resistance-compliance network model [17] or an electrical circuit as an analogue to the ventilator-circuit-patient [12, 18] have also been proposed by other groups. In addition, in [3] a criteria to select patients to be connected to the naive dual ventilation system has been proposed based on numerical models, the required level of support on the lower PEEP/higher FIO_2_ and values of lung compliances and resistances.

None of these works have analysed in detail the exhalation dynamics. The estimation of the exhalation characteristic times with linear lumped element models, as considered in these works, takes a simple form when two identical patients are ventilated under the same conditions and with the same exhalatory circuits. However, these ideal cases are the exception. As we will show, when asymmetries of expiratory limbs or patients’ tidal volumes occur, no simple expression of the characteristic times can be derived with these traditional analogous electric models. At the same time, there are some limitations of the linear lumped element models that have been discussed previously by different authors (see, for instance, [19]). Particular drawbacks of these approaches are that they consider linear behaviours of the different circuit parts and that distributed elements may behave differently from lumped elements.

Hence, to adequately evaluate the coupled dynamics of exhalations, the development of a refined model seems necessary. In brief, this work aims to analyse the potential adverse interactions during exhalation of mismatched patients connected in parallel to a shared ventilator device with individual regulation of PIP and PEEP. The study is based on results issued from a validated numerical model that we have developed specifically for this purpose.

The rest of this article is organized as follows. In section 2 we describe the model and its validation procedure. In section 3 we show and discuss results considering an ACRA device and a range of compliances and resistances of patients compatible with Acute Respiratory Distress Syndrome. Then, in section 4 we discuss the limitations of our model. Finally, we summarize our main findings in section 5.

## Methods

Experimental and numerical tests were carried out to study the possible interaction effects between patients of a shared pressure-controlled ventilation device. While a numerical model is a convenient tool that can accurately characterize the behaviour of many complex system, its performance must be validated against experimental tests. Hence, we first present the laboratory benchmark that allowed the validation and calibration of the model which is analysed afterwards.

### Experimental setup

Dual ventilation experimental data was acquired using an ‘ACRA’ device that was connected to an intensive care unit (ICU) ventilator in pressure-controlled mode. By using valves the device allows accurate control in inspiratory and expiratory pressures on both patients.

As opposed to a system in which a single patient is connected to a ventilator, the inspiratory pressure and PEEP are not directly read from the ventilator monitor. These values are determined by means of setting parameters in the ventilator monitor and manipulating the adjustable valves of ACRA within each patient’s circuit. This enables the user to independently modify patient driving pressures.

In this study we used two ACCU LUNG Precision Test Lungs (Fluke Biomedical) and two SmartLung 2000 (IMT analytics) lungs to mimic patients. Also, measurements of pressures and flow rates were acquired through orifice plate differential pressure sensors and a FluxMed monitor (MBMed).

**Fig 1:**
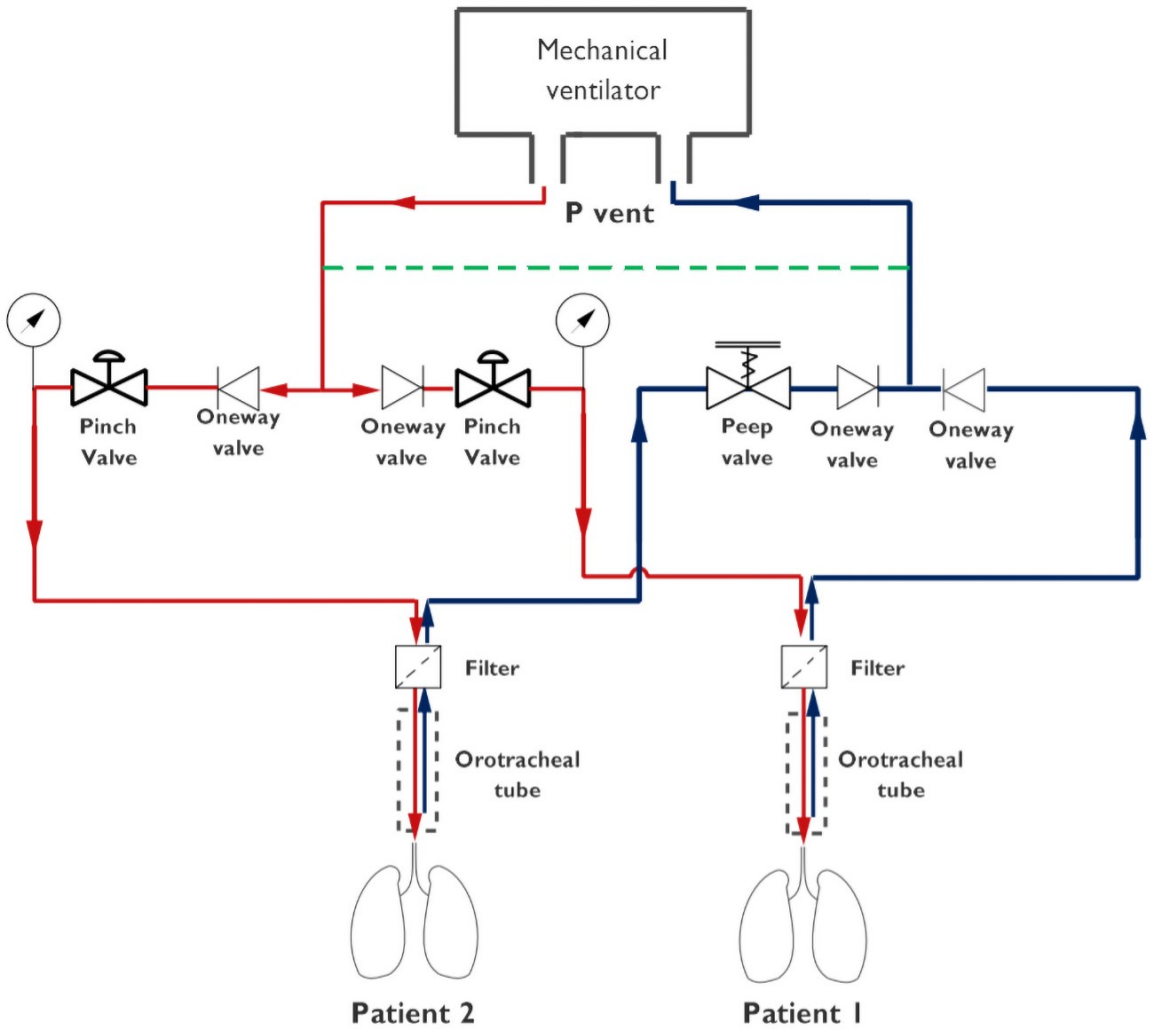
Basic single-line scheme of the two-compartment model. Inspiration and expiration circuits are coloured in red and black respectively. A dashed green line represents a short-circuit tubing of small diameter.

A simplified diagram of the experimental set up is shown in Fig 1. Further constructive details of ACRA can be found in Appendix A.

**Fig 2:**
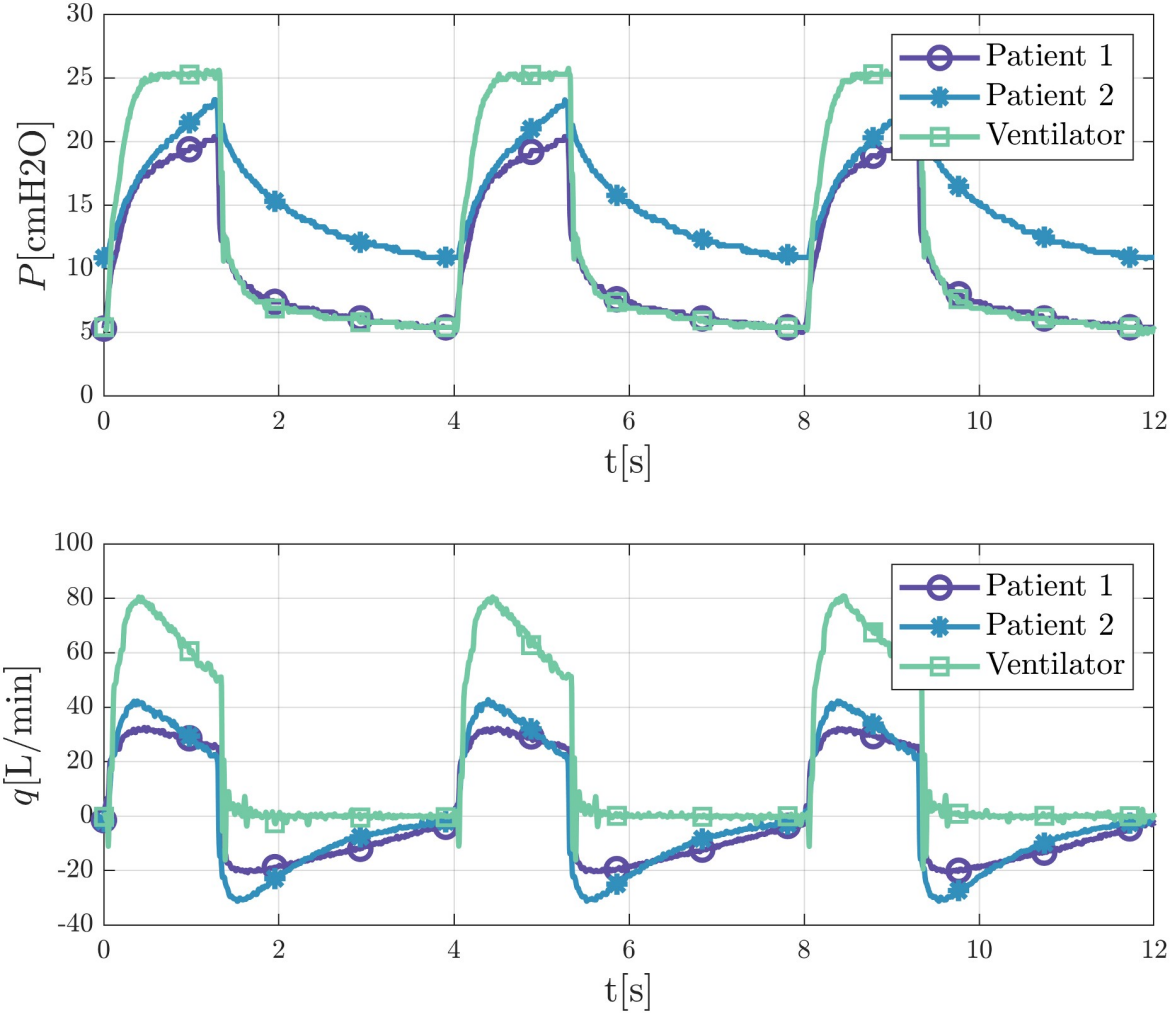
Typical output for pressure and flowrate signals of the ventilator and of artificial lungs probes with different working parameters.

Typical experimental output signals of pressures and flowrates are presented in Fig 2. Probes placed close to the ventilator and close to each mechanical lung allow to characterize the main fluid dynamics behaviours of the inspiratory and expiratory cycles.

While keeping the output of the ventilator constant and adjusting the constriction of pinch valves or the in-line PEEP valve, different signal waveforms can be observed. When closing the gap of the adjustable pinch valves, pressure values at the end of the inspiration decrease, meanwhile turning the knob of the in-line PEEP modifies the signals of expiratory pressure.

Fig 2 shows that waveforms of lung pressure do not achieve the plateau pressure of the ventilator output. The same Figure also illustrates that the flowrate signals exhibit a decelerating flow waveform pattern with a nonzero value at the end of inspiration. The existence of this nonzero flow value at the end of the inspiration and the associated pressure drop produced by its passage through the valve explains why the pressure does not attain the plateau pressure.

**Fig 3:**
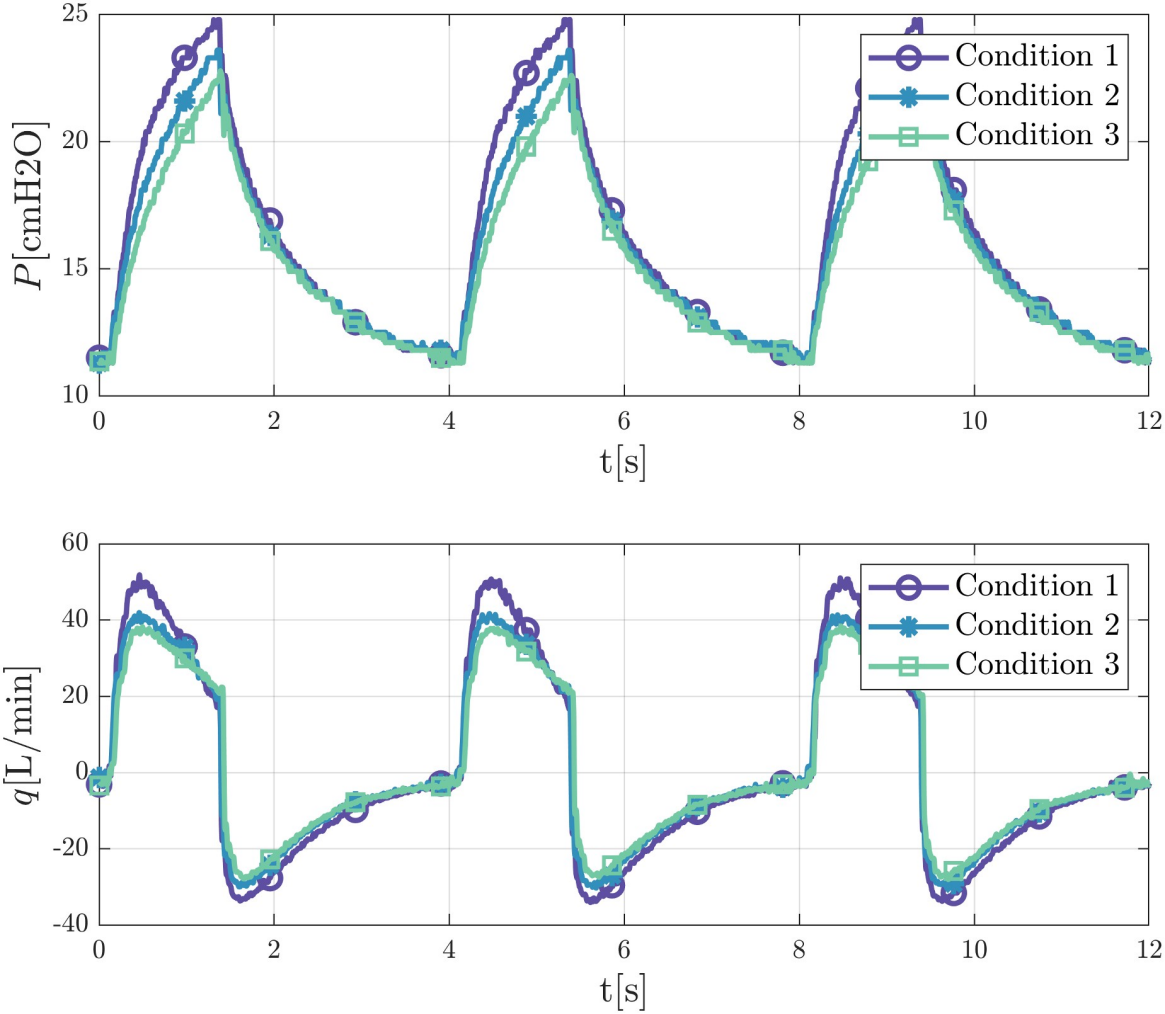
Different pressure and flow rate signals of a patient for a fixed ventilator output. Valve closes gradually from condition 1 to condition 3. Measurements at the pressure port of the test lung.

Fig 3 illustrates the effect of adjusting the constriction gap of one patient pinch valve when keeping the output of the ventilator constant. We observe that by closing this gap waveforms are modified, and the pressure at the end of the inspiration decreases. The Figure shows that closing the gap not only modifies the inspiration curves, as would be expected, but also the exhalation curves as a consequence of alterations of the delivered tidal volume to the patients. In this work, we will focus our study on these dynamics through the models we next describe.

### Numerical model

**Fig 4:**
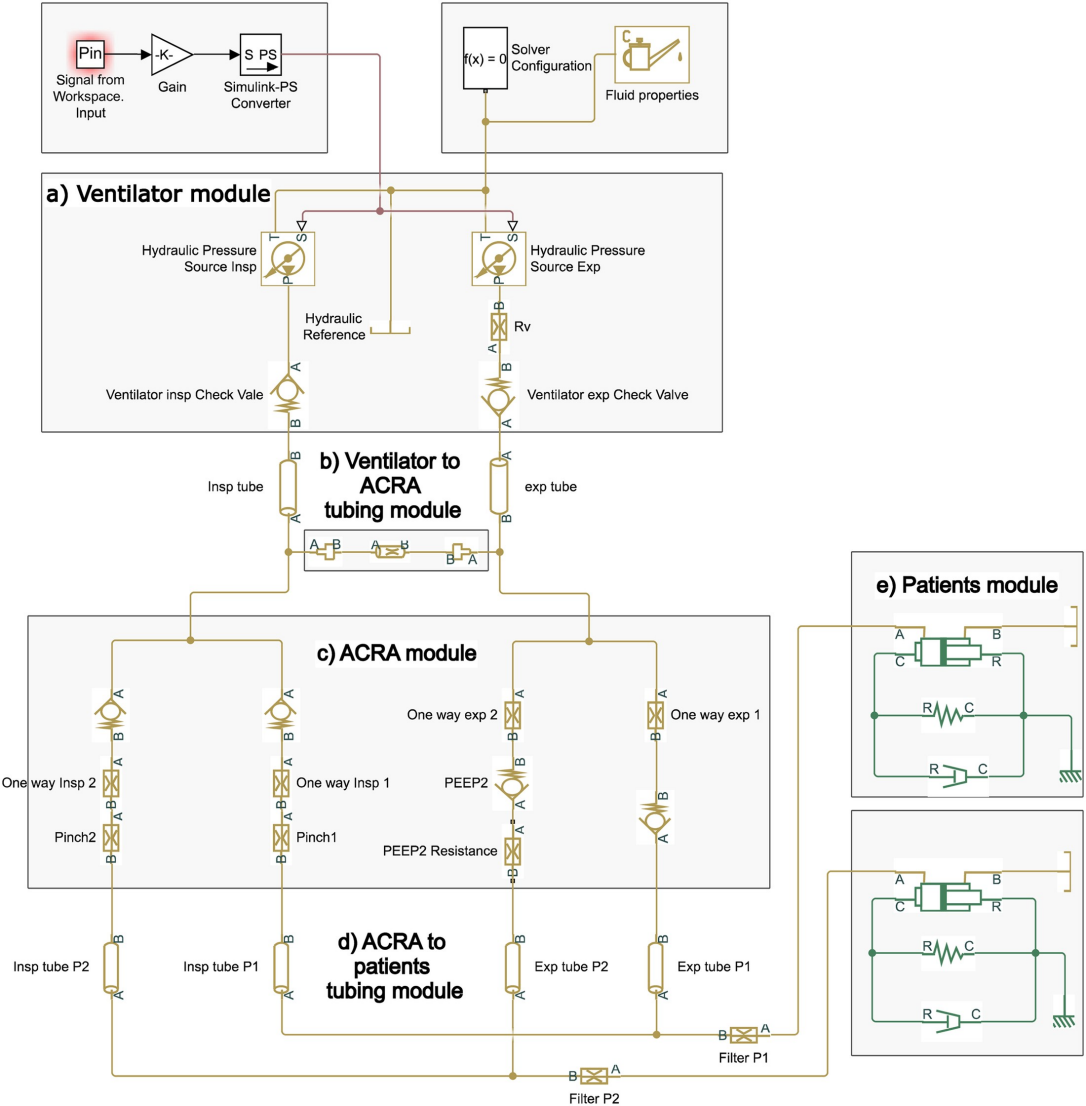
Simulink scheme of the ACRA circuit.

The numerical model we next describe enables us to undertake a comprehensive parametric study of patient conditions and ventilatory requirements to reveal in which situations exhalation dynamics of patients may mutually interfere, producing auto-PEEP in any of the convalescent persons. This model of the circuit was developed with Matlab Simulink using Hydraulic and Mechanical libraries. The code we developed with this platform is open and available on [https://github.com/ACRA2020]. Simulink is a data flow graphical programming language tool for modelling, simulating and analyzing multi-domain dynamic systems, and the block diagram considered is sketched in the comprehensive scheme presented in Fig 4.

The solver used in Simulink was ode23t. The trapezoidal rule is implemented in this solver to integrate the ordinary differential equations. The method features a variable step, and a relative tolerance of 1E-3 was stipulated. It is important to mention that this method is more appropriate than Runge-Kutta methods (as implemented in ode45) when the problem is stiff, which happens to be the case due to the high temporal gradients imposed by the ventilator pressure curves used as inputs. Similar strategies have been used by other authors analysing dual ventilation systems [12, 17]. Our scheme uses the circuit of an ACRA system but can be easily adapted to other dual ventilation circuits. It comprises: (a) the ventilator, (b) the common circuit parts: inspiratory, expiratory and short circuit tubing, (c) the individualized inspiratory flow circuit, (d) the individualized expiratory flow circuit, and (e) the patient 1 and 2 mechanical lungs models respectively.

**Fig 5:**
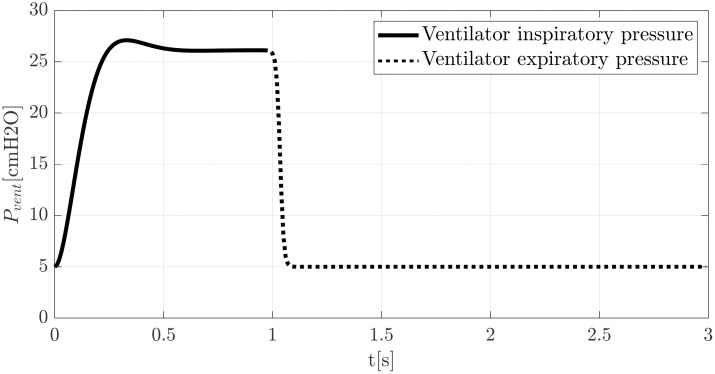
Typical waveforms of pressure signals for inspiratory and expiratory cycles.

**Fig 6:**
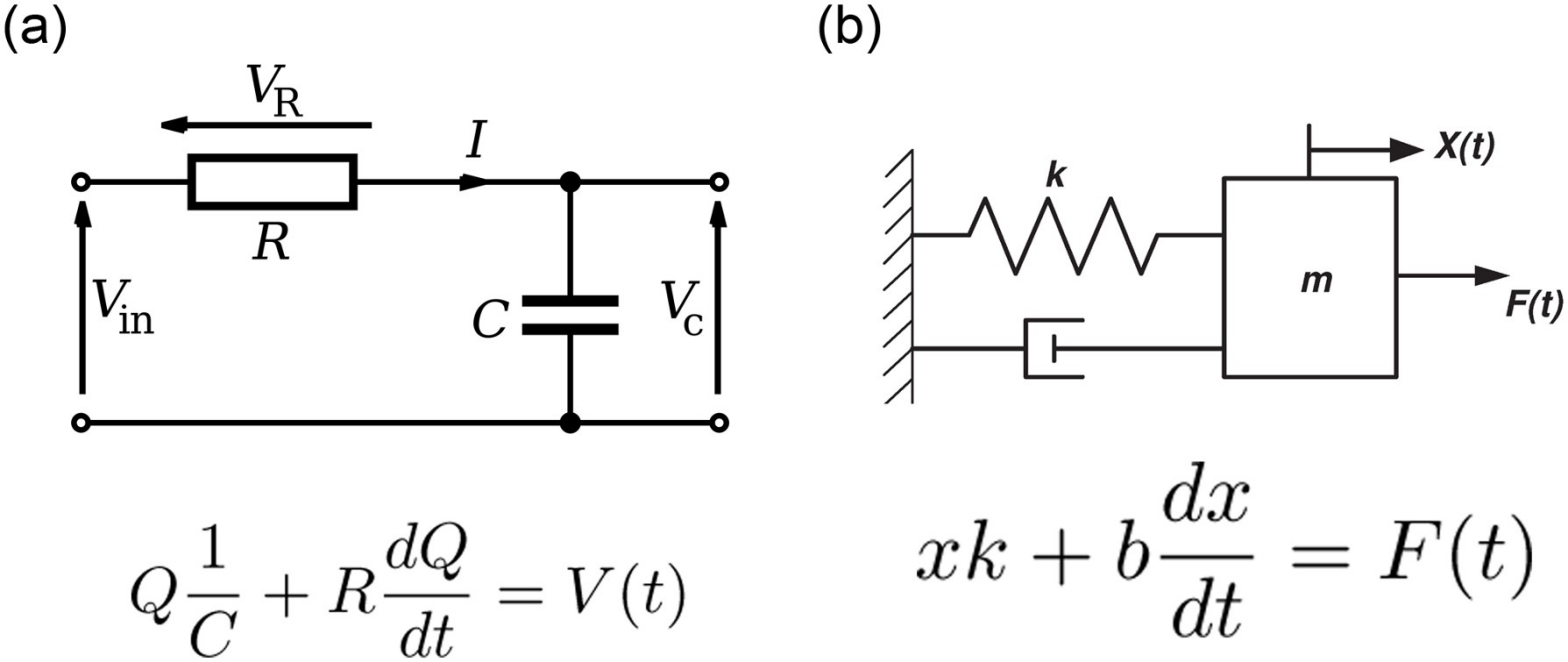
Equivalence between (a) RC model and (b) Mechanical model. The electric charge (*Q*) and current (*dQ*/*dt*) are equivalent to the position (*x*) and velocity (*dx*/*dt*) respectively. By inspection, *k* ≡ 1/*C* and *b* ≡ *R*. In hydraulics terms, flow rate ${\dot{V}}_{L_{i}}\equiv dx/dt$ and flow volume $V_{L_{i}}\equiv x$.

The main features of the model were estimated assuming the experimental values taken from the ACRA description presented in Appendix A. The circuit is composed of:

(a) Ventilator Module: Fluid properties and a periodic pressure signal define two hydraulic pressure sources, which account for inspiratory and expiratory phases. The input signal consists of an underdamped function for the inspiration and a decreasing sigmoid function for the expiration respectively, as depicted in Fig 5. The ventilator sources are followed by check valves to guarantee the correct sense of the flow during inspiration and expiration cycle. A flow resistance is used to consider the effect of flow restriction of the exhalatory valve.
(b) Ventilator to ACRA Tubing Module: To make compatible the functioning of ACRA device with any Ventilator closed loop control system, a flexible silicon short-circuit tube is introduced between the inspiration and expiration branches. Its diameter and length are *D*_*sc*_ = 4 mm and *L* = 15 cm respectively, with its head loss modelled by a resistive hydraulic pipeline element. Hydraulic pipelines of the model incorporate not only head losses but also fluid structure-interaction (compliances) of circuit tubes. The model uses the Darcy equation, where head losses are proportional to a flow regime-dependent friction factor *f* and the square of the flow rate ${\dot{V}}_{L_{i}}$.
  $$\begin{array}{c} \Delta P=f\left(Re\right)\frac{L}{D}\frac{\rho }{2A^{2}}{\dot{V}}_{L_{i}}^{2} \end{array}$$ (1)
  Where, *A* is the pipe cross-sectional area, *D* the Pipe hydraulic diameter, *L* the pipe geometrical length and *ρ* the fluid density. As *f* also depends on roughness, *f* = *f*(*Re*, *ε*/*D*), we considered *ε* ≃ 0.2 mm. The compliance of the tubings was considered assuming elastic flexible pipe walls. Considering that in a circular pipe $\frac{dV}{dP}=\frac{L\pi D}{2}\frac{dD}{dP}$ where *D* = 20 mm and *L* = 1 m, and using typical values of tube compliance *dV*/*dP* = 1 ml / cm H_2_O [20] static pressure-diameter coefficient was determined *dD*/*dP* ≃ 4 ⋅ 10^−8^ m Pa^−1^. The length and internal diameter of the tubes used in this module are *L* = 1 m and *D* = 20 mm respectively.
(c) ACRA Module: A bifurcation of the inspiratory circuit of the ventilator determines in the ACRA two different branches. Each branch has a one-way valve to force the adequate flow sense and a pinch valve that controls the flow rate. Pressure losses generated by these elements were computed using localized flow resistances through:
  $$\begin{array}{c} \Delta P_{i}=K_{i}{\dot{V}}_{L_{i}}^{2} \end{array}$$ (2)
  *K*_*i*_ is the localized loss coefficient for each element, which is assumed to be constant and experimentally determined (see Appendix B).
  The expiratory circuit is similar to the inspiratory one. The difference between them is that an external PEEP or threshold valve (modelled with a check valve) was used to control the PEEP value for patient 2. The pressure loss generated by the PEEP valve was modelled using Eq (2), where *K*_*i*_ = *K*_*PEEP*_ is the PEEP valve loss coefficient experimentally determined (see Appendix B).
(d) ACRA to Patients tubing Module: This module is similar to the Ventilator to ACRA Tubing Module. The length and internal diameter of the tubes used in this module are *L* = 1 m and *D* = 20 mm respectively.
(e) Patients Module: A mechanical system that consists of a spring, a damper and a piston was used to represent the lungs of patients 1 and 2 respectively. As they are often studied under electrical model analogies, the equivalence of both approaches is illustrated in Fig 6. Let us remark that we introduced nonlinear laws for the spring and damper elements, attending the particular mechanical lungs properties we used in the experiments (see Appendix B). Position and velocity of the piston (mechanical parameters) correspond to volume displacement and flow rate on the hydraulic circuit.

In Appendix B we include further details on how the different model parameters are determined.

**Fig 7:**
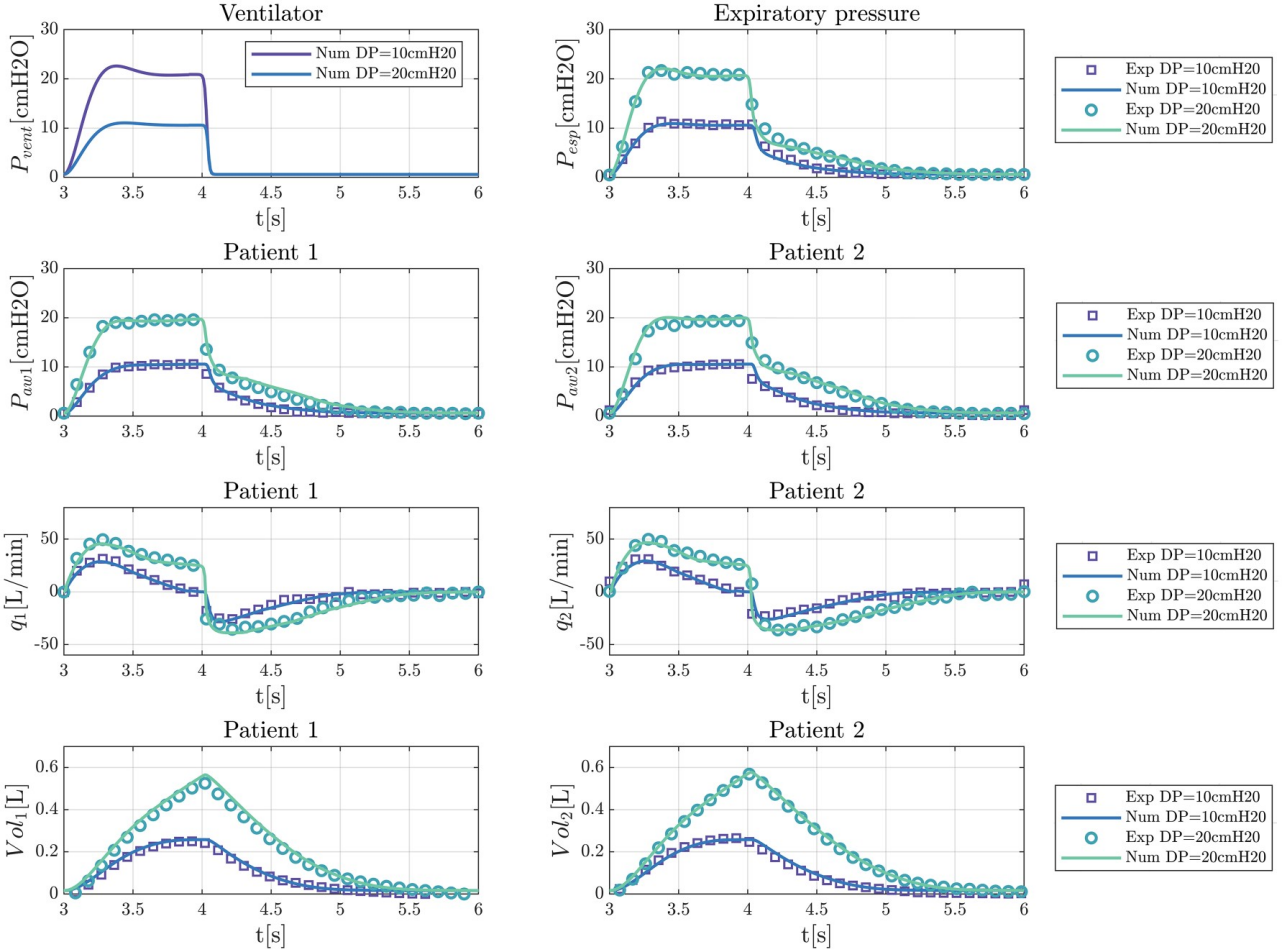
Validation tests for two driving pressures.

In Fig 7 we show a validation of the model with typical results for tests performed for two different driving pressures, 20 and 10 cm H_2_O. We confirm a remarkable agreement between the data obtained with the model and measurements.

## Results and discussion

The characteristic exhalation times of each patient (*τ*_*i*_) are used to analyse how the volume of gas in the lungs evolve with time when the fluid is released. When exhalation follows an exponential decay with time, 4*τ*_*i*_ corresponds to the amount of time needed to empty 98% of the volume. Considering that the available time for the expiratory cycle is *t*_*ex*_ = 1/*BR* − *t*_*ins*_ (with breathing rate *BR* expressed in min^−1^) and that to empty the lungs of patient *i* the time required is ≃4*τ*_*i*_, the result is that to avoid auto-PEEP 4*τ*_*i*_ ≤ (1/*BR* − *t*_*insp*_). In other words, if the ratio 4*τ*_*i*_/*t*_*ex*_ is greater than 1, the lungs will not be fully empty at the end of expiration and auto-PEEP phenomenon may occur. For the sake of clarity, we will use this nondimensional ratio defined as $\tau_{i}^{*}=4\tau_{i}/t_{ex}$ to analyse the characteristic times of the patients during the expiration cycle.

To analyse exhalatory dynamics, it is typical to consider an electric analogy with linear lumped elements in which volume is associated with electric charge, flow rate to electric current, pressure to voltage, friction losses to electrical resistance, and compliance to capacitance.

In Appendix C a simplified linear lumped element model is presented. In this case, some basic assumptions such as neglecting pressure losses generated on the one-way valves, on the PEEP valve and on the tubing are made. Also, tube compliances effects are not considered in this simplified model.

A refined model including a linearization of resistances and compliances of the missing components of the circuit, does not necessarily improve the accuracy in predicting time scales. This is due to the high nonlinearities of the components and to the large range of volumes and flows involved in the analysed phenomena.

Taking into account these considerations, the simplified analytical model proposed should be considered as a simple tool that provides reference values and that helps to unveil some relevant aspects of the exhalation dynamics in some specific situations.

For instance, the characteristic exhalation time can be easily estimated when both patients’ respiratory parameters, circuit, and driving pressures are identical and the exhalatory valve flow resistance is negligible. In this case, the expiratory dynamics of both patients are coincident and the characteristic time can be determined with the conventional expression *τ*_*A*_ = *RC* where we denoted with *R* the total resistance of patient and expiratory circuit, and *C* the total compliance (sub-index ‘A’ refers to analytical solution).

When incorporating the effect of the exhalatory valve flow resistance in the analysis for these patients, it is easy to demonstrate that *τ*_*A*_ = (*R* + 2*R*_*V*_)*C*. Note, that this time is larger than the one where the patient is treated individually with the same ventilator (*τ*_*A*,*I*_ = (*R* + *R*_*V*_)*C*) (sub-index ‘I’ refers to individual ventilation). In consequence, connecting identical patients in parallel will always increase the time required for patients to empty the lungs, even in the quite favourable situation of identical patients. In general, when asymmetric situations take place, only a lower limit can be obtained for each patient considering uncoupled dynamics with *τ*_*A*,*i*_ ≥ *R*_*i*_ *C*_*i*_ for patient *i*.

A simple nondimensionalization of the simplified model equations, presented on Appendix C, indicates that the nondimensional characteristic times of each patient can be expressed in terms of:

$$\begin{array}{c} \tau_{i}^{+}=f\left(\frac{R_{2}}{R_{1}},\frac{C_{2}}{C_{1}},\frac{R_{V}}{R_{1}},\frac{DP_{2}}{DP_{1}},\frac{{PEEP}_{2}}{{PEEP}_{1}}\right) \end{array}$$ (3)

where *R*_*i*_ and *C*_*i*_ represent the airway resistance and lung compliance of patient i (assumed constant and independent of flows and volumes), *R*_*V*_ the ventilator resistance, *PEEP*_*i*_ the PEEP value of patient i, and *DP*_*i*_ the *exhalatory driving pressure* defined as *DP*_*i*_ = *P*_*i*_ − *PEEP*_*i*_ (where *P*_*i*_ is the pressure of patient i at the initiation of exhalation). This pressure difference is intimately related to the traditional *driving pressure*, more popularly known in the context of mechanical ventilation inspiratory dynamics.

The limitations of the analytical solutions do not allow to accurately determine the times required for exhalation. For a given ventilator and circuit, one possibility to determine the functions of Eq (3) could be to rely on an experimental approach. However, the number of tests to be performed would be very large.

Therefore, in this context, the use of numerical modelling appears as an interesting and versatile tool to perform the parametric analysis we next describe.

### Numerical estimation of characteristic times

In this section, we propose to numerically evaluate the incidence of the different ratios of Eq (3) on the characteristic exhalation time of each patient. For this purpose, we apply the discussed numerical model, and for each test, the ventilator parameters were set to the typical values presented in Table 1.

**Table 1:** Operating conditions for the ventilator.

Breathing rate	*BR* = 18min^−1^
Inspiratory time	*t*_*ins*_ = 1 s
PEEP value	*PEEP* = 5 cm H_2_O
Maximum inspiratory pressure	*P*_*max*_ = 32 cm H_2_O

In all calculations, the driving pressure of patient 1 was set to a fixed value of *DP*_1_ = 15 cm H_2_O and resistance and compliance for patient 1 were respectively *R*_1_ = 5 cm H_2_O (L/s)^−1^ and *C*_1_ = 35 mL (cm H_2_O)^−1^.

Three combinations of driving pressures (*DP*_2_/*DP*_1_ = [0.75 1.00 1.50]) for three combination of PEEP values ([*PEEP*_2_/*PEEP*_1_ = [1.0 1.5 2.0]) were simulated. The patient 2 resistance values considered were in the range of 2 to 8 cm H_2_O (L/s)^−1^ and compliance values between 27.5 and 42.5 mL (cm H_2_O)^−1^. Consequently, the ratios *R*_2_/*R*_1_ lay within the range of 0.40 to 1.21 and *C*_2_/*C*_1_ in the range of 0.80 and 1.21.

The numerical characteristic times *τ*_*N*,*i*_ (sub-index ‘N’ refers to numeric and sub-index ‘i’ refers to patient ‘i’) were estimated by fitting the results of tidal volumes with time, with an exponential decay function $VT_{i}\left(t\right)=a+be^{-t/\tau_{N,i}}$ where sub-index ‘i’ = 1,2 indicates patient number and *a* and *b* are the fitting parameters.

The results of the linear lumped element model suggests that in real systems, the exhalation should be expressed with a series of exponential functions (see Eq (8)). However, the results of the numerical model shows that this refinement seems unnecessary to capture the essential physics of the problem. With this model for all the studied cases, the fitting of the results with a single exponential function gave a coefficient of determination *R*^2^ higher than 0.99. Hence, we consider that the use of a single time constant for each patient is representative enough to describe the involved exhalatory dynamics.

In the next sections, a parametric study of the characteristic times for the different ratios of parameters will be discussed. To this end, we will consider the nondimensional ratio calculated using either the numerical model $\tau_{N,i}^{*}=4\tau_{N,i}/t_{ex}$ or the simplified linear lumped element model $\tau_{A,i}^{*}=4\tau_{A,i}/t_{ex}$.

#### Identical patients with identical ventilation conditions

**Fig 8:**
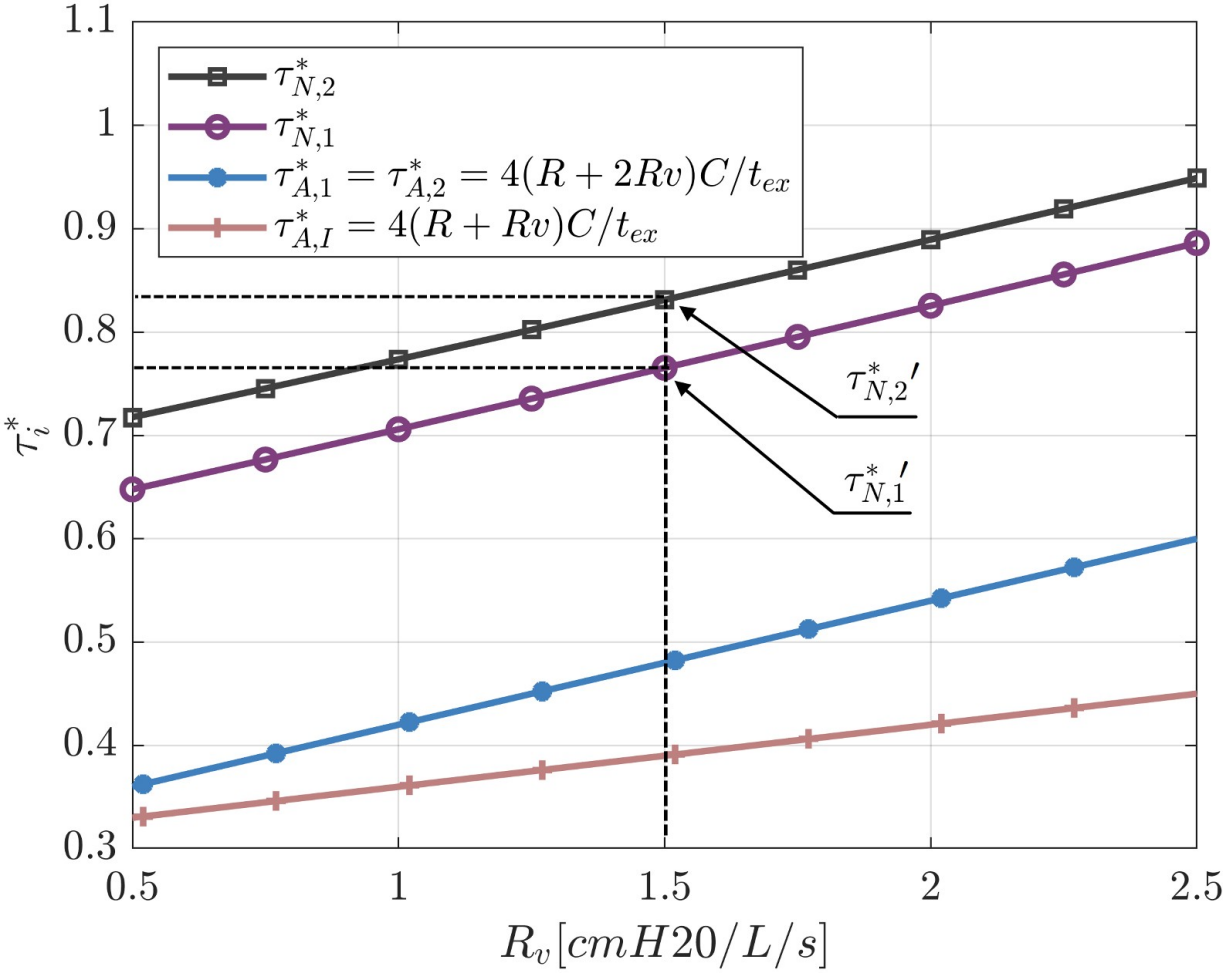
Influence of exhalatory valve resistance on characteristic times for symmetric conditions (*PEEP*_1_ = *PEEP*_2_ = 5 cm H_2_O, *DP*_1_ = *DP*_2_ = 15 cm H_2_O, *R*_1_ = *R*_2_ = 5 cm H_2_O (L/s)^−1^, *C*_1_ = *C*_2_ = 35 ml (cm H_2_O)^−1^ and *K*_*PEEP*_ = 0.0012 cm H_2_O (L/min)^−2^.

In this section two identical patients with the same conditions of ventilation will be analysed. Fig 8 shows the influence of the ventilator exhalatory resistance on the characteristic times of the patients. In this Figure the ventilator resistance *R*_*V*_ = 1.5 cm H_2_O (L/s)^−1^ of our system is marked by the dashed line and serves to determine two reference values ${\tau_{N,1}^{*}}^{\prime }$ and ${\tau_{N,2}^{*}}^{\prime }$. As expected, an increase in the ventilator exhalatory resistance results in an increase of the characteristic times for both patients. We can observe that even though patients have the same compliance, resistance, ventilation conditions, and identical circuit check valves, their characteristic times are different. This is because the presence of the PEEP valve on one of the patients’ expiratory branch. This valve generates an asymmetry of the circuits and therefore differences on the characteristic time ratios. The associated valve loss coefficient in the system considered is *K*_*PEEP*_ = 0.0012 cm H_2_O (L/min)^−2^. This in-line valve introduces a pressure loss in the circuit of patient 2 even when adjusted to 0 cm H_2_O and therefore not altering the PEEP value established from the ventilator.

It is of interest to compare these results with the characteristic times when a single patient is individually connected to the ventilator (estimated with *τ*_*A*,*I*_ = (*R* + *R*_*V*_)*C*) and with the one provided by the linear lumped element model (estimated with *τ*_*A*_ = (*R* + 2*R*_*V*_)*C*).

**Fig 9:**
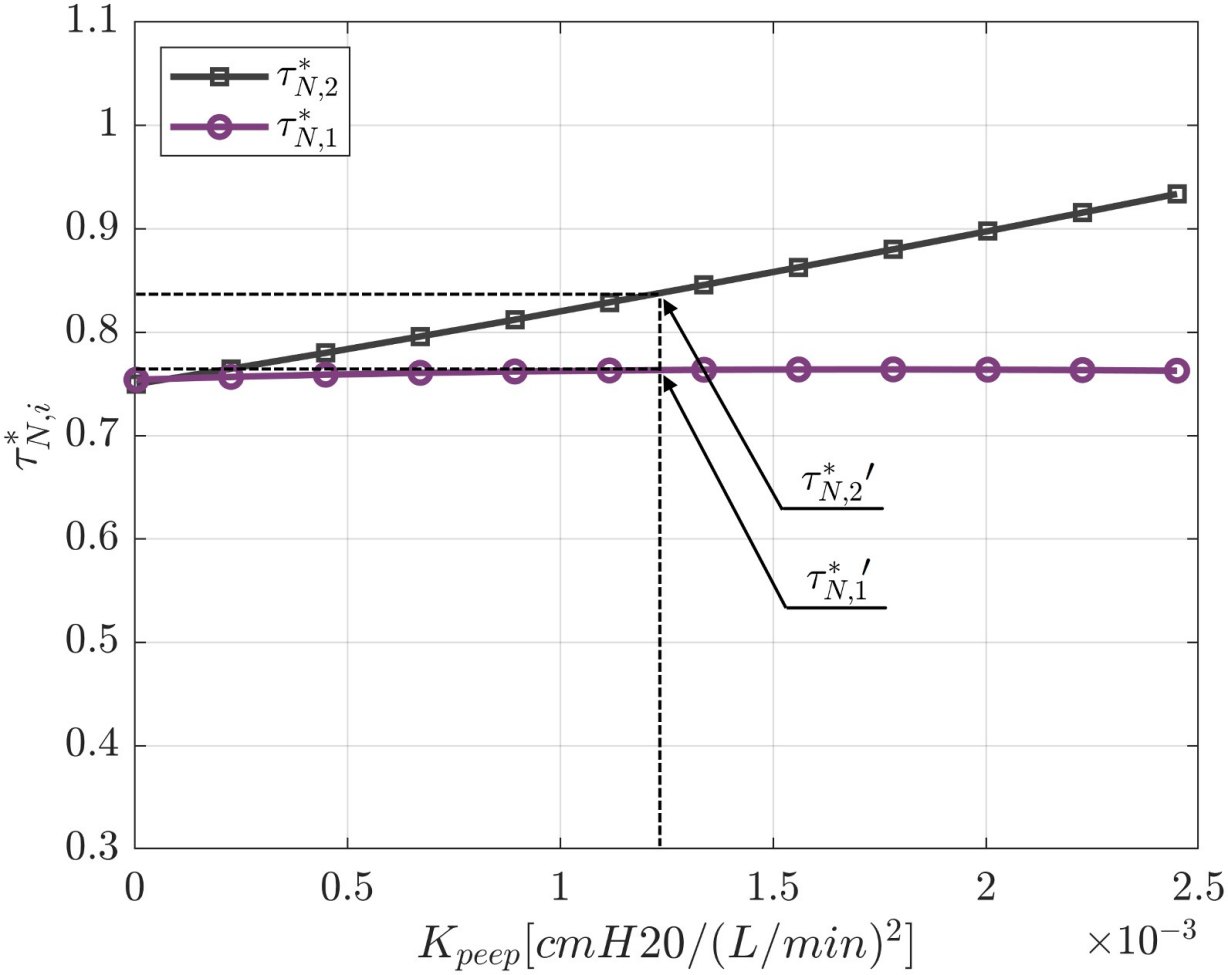
Influence of in-line PEEP valve resistance on characteristic times for symmetric conditions (*PEEP*_1_ = *PEEP*_2_ = 5 cm H_2_0, *DP*_1_ = *DP*_2_ = 15 cm H_2_0,*R*_1_ = *R*_2_ = 5 cm H_2_0 (L/s)^−1^, *C*_1_ = *C*_2_ = 35 ml (cm H_2_0)^−1^), and *R*_*V*_ = 1.5 cm H_2_0 (L/s)^−1^.

As we can see in Fig 9, the characteristic time ratios $\tau_{N,1}^{*}$ and $\tau_{N,2}^{*}$ are much higher than the characteristic time when only one patient is connected to the ventilator. The reason for this result is that when two patients are connected to the ventilator, the pressure loss on the ventilator exhalatory valve increases drastically due to the rise of the total flow (now $\dot{V}=\dot{V_{1}}+\dot{V_{2}}$ and $\Delta P_{v}=R_{V}\dot{V}$). However, this effect may be largely mitigated when exhalatory valves of the ventilator exhibit lower values of flow resistance.

Fig 9 shows the influence of the PEEP valve resistance on the characteristic times ratio. When the PEEP valve resistance is negligible, the symmetry is complete and $\tau_{N,1}^{*}=\tau_{N,2}^{*}$. However, as the PEEP valve resistance increases, patient 2 shows an increase in $\tau_{N,2}^{*}$ or, in other words, there is more restriction on the patient’s ability to expel air from the lungs. In contrast, the effect on patient 1 characteristic time is negligible (its value remains almost constant for the simulated cases).

#### General case

**Fig 10:**
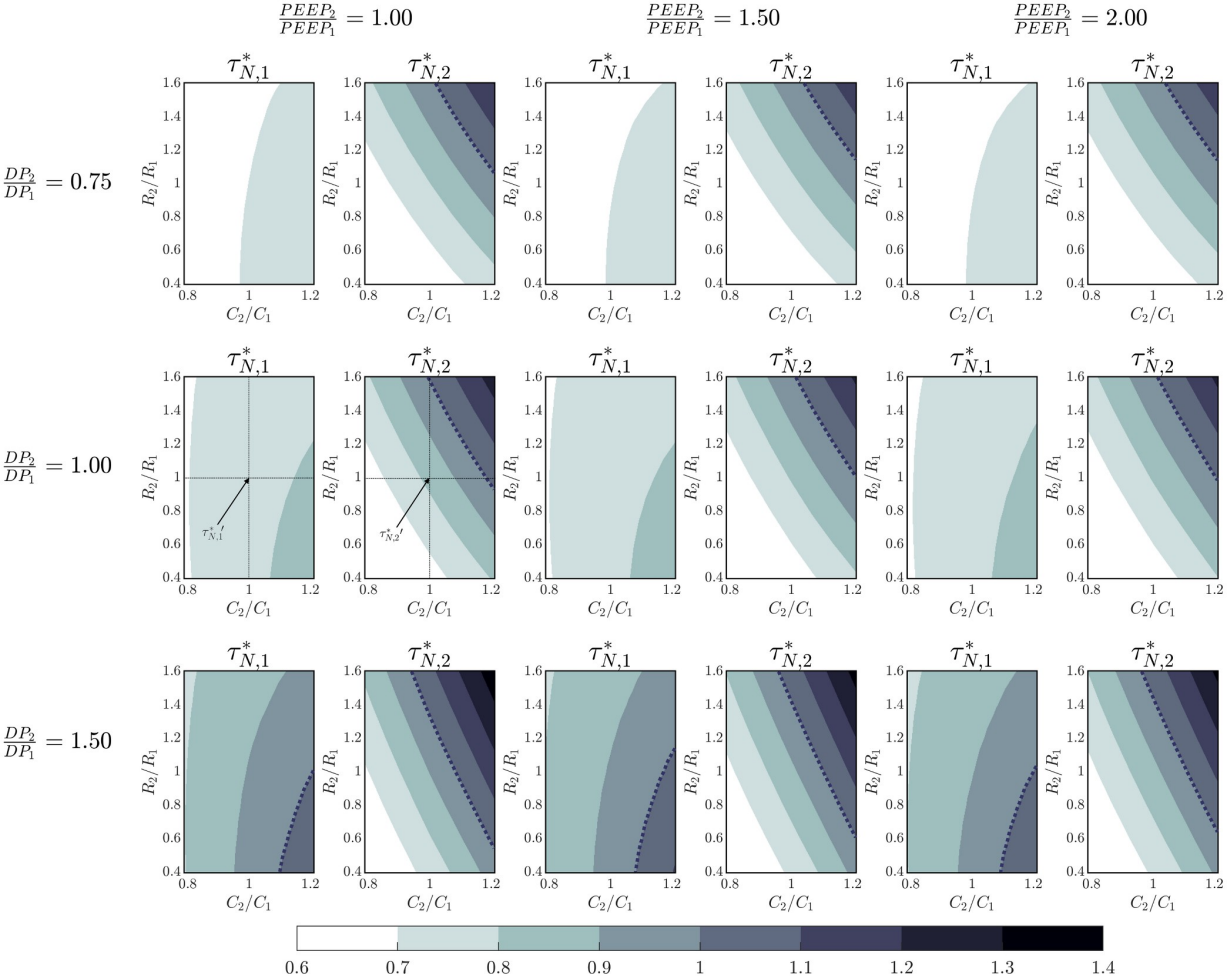
Characteristic exhalation times mapping for *R*_*V*_/*R*_1_ = 0.3 and for different patients’ lungs mechanics combinations. The dashed line represents the auto-PEEP limit.

In Fig 10, the characteristic time ratios $\tau_{N,1}^{*}$ and $\tau_{N,2}^{*}$ for different PEEP ratios (columns) and driving pressure ratios (rows) are presented using a colour map for a *R*_*V*_/*R*_1_ ratio equal to 0.3. A dotted line is also depicted in each Figure separating the auto-PEEP region (characteristic time ratio greater than 1) and the non auto-PEEP region. Also, the same $\tau_{N,1}^{*}\prime$ and $\tau_{N,2}^{*}\prime$ shown in Figs 8 and 9 are marked on Fig 10 (symmetric case).

The analysis of these Figures indicates that two identical patients (same resistance and compliance) ventilated with different parameters (driving pressure or PEEP) do not suffer from auto-PEEP. Auto-PEEP is more probable to occur when patients are different. In this case, depending on the ratio of driving pressure and the asymmetry of patients, auto-PEEP may take place in one or in both patients. The patient with the in-line PEEP valve is more prone to auto-PEEP than the other. The auto-PEEP of any patient is not quite sensitive to the ratio of PEEP considered. The risk of auto-PEEP for both patients is higher when the compliances of the second patient are higher. The risk of auto-PEEP also increases when the ratio *R*_*V*_/*R*_1_ becomes greater for every combination of patients and ventilation parameters.

To characterize the changes produced on the characteristic times due to asymmetries of the delivered driving pressure, it is convenient to consider the coefficient $\tau_{N,i}^{*}/\tau_{N,ref}^{*}-1$ (with $\tau_{N,ref}^{*}$ the case corresponding to equal driving pressures).

**Fig 11:**
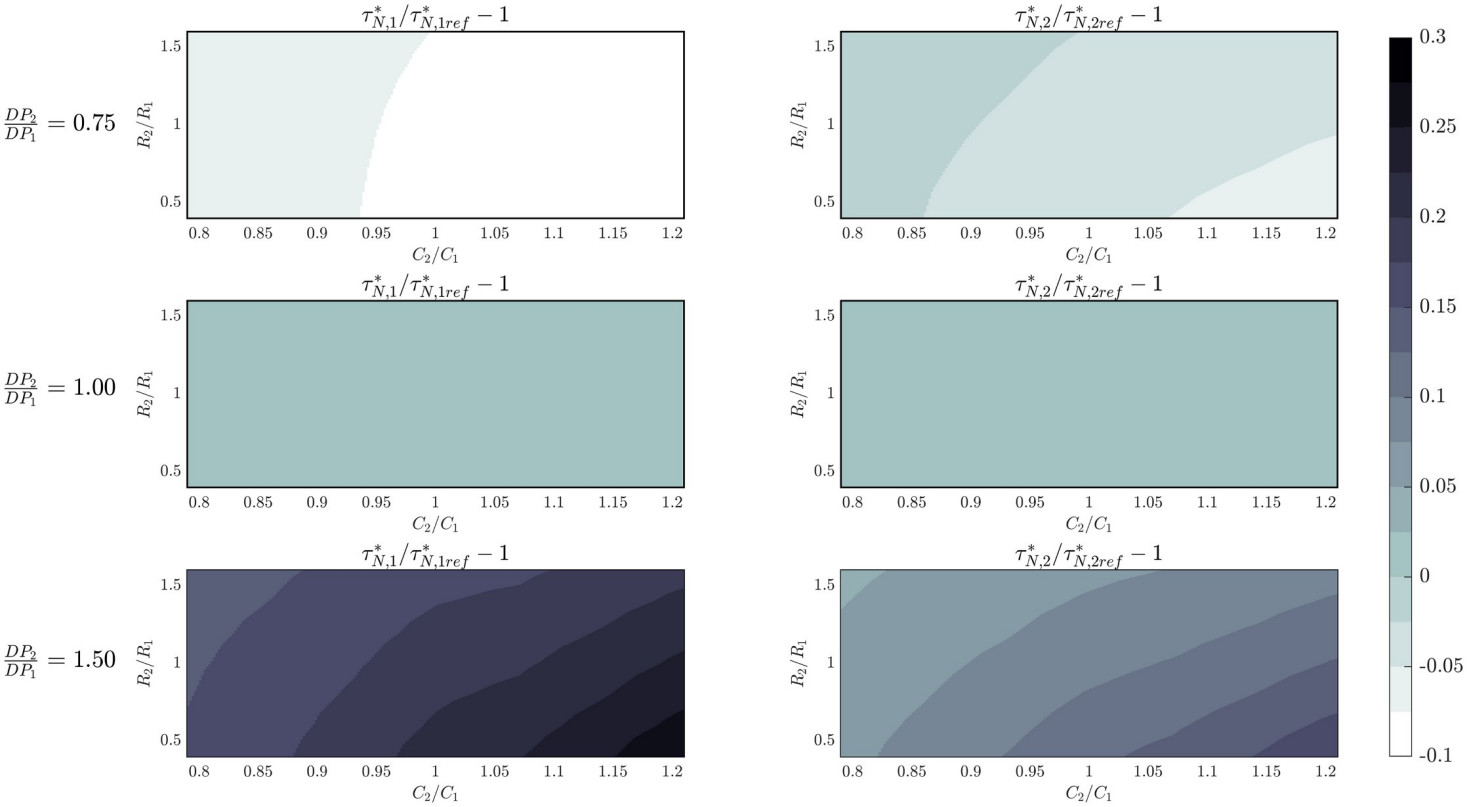
Incidence on exhalation characteristic times of asymmetries between patients.

Fig 11 depicts the values attained by this coefficient for different resistance, compliance and driving pressure ratios for *R*_*V*_/*R*_1_ = 0.3. We included in the second row the case of equal driving pressures as a reference (in this case, the values of the coefficient are constant and equal to 0). The first row of the Fig 11 shows the case of a 25% decrease on driving pressure 2 and the third row the influence of a 50% increase on driving pressure 2. In all these cases it can be observed that the asymmetries of driving pressure significantly alter the characteristic time of both patients. The effect is more pronounced for the patient with the lower driving pressure. These results can be explained considering that when driving pressure is increased, tidal volume also increases. Consequently, the combined effect of pressure losses and the coupling associated with flows gathering at the node result in more difficulties to empty the lungs of the patients.

### Relevance of this study to clinical guidelines

Regulation Agencies and Medical associations in different countries have stated that alternative ventilation devices proposing pressure control modes of ventilation should be able to control PIP and PEEP [12, 21]. Taking into account these requirements, strategies of dual ventilation based on the use of simple splitters (naive sharing circuits devices) have been legitimately questioned because mismatch of resistances and compliances may lead to inadequate ventilation of patients. However, previous works have shown that incorporating to the naive sharing circuit devices variable resistances, it is possible to individualize the tidal volume of each patient through controlling the specific PIP delivered to each patient. Furthermore, by incorporating in-line PEEP valves, it could also be possible to individualize the control of PEEP delivered to each patient. However, this last control may also depend on an adequate matching of patients because of possible auto-PEEP. Our study shows that the risk of auto-PEEP is quite limited for identical patients. For similar patients, to mitigate the possibility of auto-PEEP, it is beneficial to connect the patient with lower compliance to the branch that incorporates the in-line PEEP valve. The larger the asymmetries in patient compliances and driving pressures, the larger the probability of auto-PEEP. Moreover, our study shows that the exhalation characteristic times of the patients is not quite sensitive to differences on the ratios of PEEP values.

## Limitations of this study

The numerical model we developed here allows the user to consider nonlinearities in compliances and resistances and shows great agreement with *in vitro* tests. However, in our study, some of these parameters like the values of exhalation resistances and compliances of each patient have been considered as constant with the flow. This may be quite simplistic. Hydraulic resistance for instance in the endotracheal tube will be constant only for laminar flow, a regime that usually takes place only at the end of the expiratory cycle. Also, the physiological relationship between pressure and flow in patients is often nonlinear (see [22, 23]). In addition, lung compliance is nonlinearly dependent on ventilation pressure due to volume-dependent tissue stiffness [24].

Taking into account previous research [25] we also considered that the resistance of the exhalatory valve was linear with the flow. Most currently available ventilators have a servo-controlled exhalation valve with a variable opening controlled by the ventilator. Usually, for a fixed position of the valve opening, the pressure drop through the valve depends quadratically with the flow $\Delta P=K_{v}{\dot{V}}^{2}$. When the valve opening changes with time, the law that links the instantaneous pressure drop with the flow can become complex over time. Particularly, the closure dynamics of the exhalatory valve may differ with the type of ventilator considered. Therefore, assuming a universal linear link between instantaneous flow and instantaneous pressure drop may also be simplistic.

The analysis we have performed considers the characteristics of the pinch valves used in the ACRA device and the characteristics of the exhalatory valve of a Nellcor Puritan Bennet 760 Ventilator. Even if the results here obtained are modified for use with other ventilators, the observed trends should be the same. However, results of this parametric study may not reflect all situations found in clinical care.

## Conclusions

In this work, we analyse for the first time the consequences in exhalatory dynamics when two patients are connected to a dual ventilation system. A simplified linear lumped element model was used to obtain reference values and to identify the set of relevant nondimensional parameters related to patients and ventilation requirements involved in the study.

We performed a parametric analysis with a computational model, distinct in its ability to include nonlinear behaviours and distributed parameters. The remarkable agreement between results issued from the model and our experimental results with test lungs enabled us to validate the model. However, the 1D model developed required an adequate evaluation of the valves’ flow resistances (circuit and ventilator) to produce reliable results. Our study shows that dual ventilation devices produce larger patient exhalation settling times than they would have with individualized ventilation. As a consequence, patients connected to shared ventilation may experience auto-PEEP. However, this effect can be mitigated with the adequate matching of patients. The presented results correspond to a specific shared ventilation device (ACRA) used with an specific ventilator (Nellcor Puritan Bennet 760). However, the developed open source code of this article, provides the opportunity to extend the analysis of auto-PEEP risk when connecting patients to other ventilators and shared ventilation devices with simple parameter characterizations. At present, our results are based on a computational model that has not been validated against any preclinical or clinical data. Hence, clinicians should not look to our study as an exact estimate of patients exhalatory dynamics.

## Appendix A: ACRA dual ventilation system description

**Fig 12:**
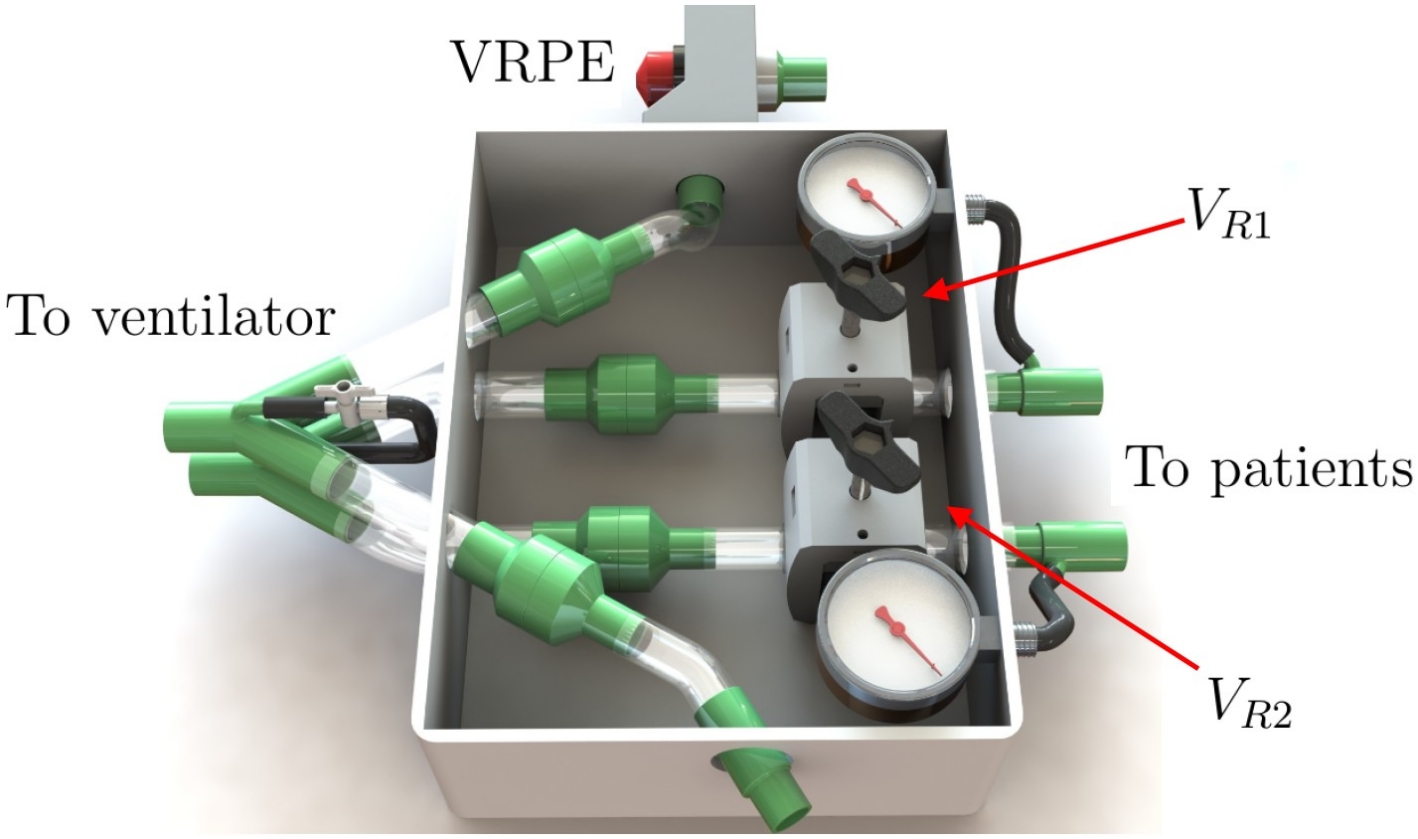
Render of the different components of the ACRA. V_*R*1_ and V_*R*2_ are flow restrictor valves (Pinch Valves). VRPE adjustable threshold valve (PEEP valve) (ad hoc design). Also shown, analogical manometers the device uses and ad hoc one-way valves (3D printed with medical-grade resin and incorporated silicone membrane).

Fig 12 shows 3D renders of the ACRA device. This apparatus has been tested in two paired breathing simulators (ASL 5000, InGMAR Medical, Pittsburgh, PA) and also with two paired pig models whose lungs were manipulated to mimic severe acute respiratory distress conditions. We performed a 2-hit experimental ARDS model. Repeated lung lavages with normal saline (30 mL kg^−1^ at 37 degree Celsius) were performed until PaO_2_/FIO_2_ = 200 mm Hg at PEEP 10 cm H_2_O. The animals were then submitted to 120 min of injurious mechanical ventilation at PEEP = 0 cm H_2_O, VT = 15 mL kg^−1^, breathing rate BR = 12 min^−1^, an inspiratory-expiratory ratio of I:E = 1/2, and a FIO_2_ = 1.0. Thereafter, baseline ventilatory settings were restored; the success of the model was confirmed with a PaO_2_/FIO_2_ = 200 mm Hg, and lung ultrasound imaging showing bilateral atelectasis in the dependent lungs.

The ACRA system incorporates 700 cm^3^ including the volume of the tubing from ventilator to the interface (300 cm^3^ = 2 × 0.4 m long 22 mm flextube). The two breathing circuits (one for each patient) incorporate 2400 cm^3^(2 × 1.6 m long 22 mm flextube breathing systems). The total volume of tubings and the ACRA system is 3100 cm^3^.

Each patient’s inspiratory pressure is established by introducing a controlled pressure drop from the ventilator output *P*_*vent*_. This pressure reduction occurs mainly in the adjustable flow restrictor (pinch valve) located on each patient’s inspiratory circuit.

Changing the value of the gap constriction modifies the flow rate/pressure drop ratio. The gap value in this constriction is adjusted manually by turning a valve knob.

The peak inspiratory pressure (PIP) in the patient is determined by the value of the flowrate at the end of the inspiratory cycle, and it will be lower than the pressure imposed by the ventilator. Therefore, the PIP is not only determined by *P*_*vent*_ and the flow restriction but also by the duration of the inspiratory cycle.

Two analog manometers are incorporated in the device to determine the pressure values downstream of each pinch valve. These devices allow to measure each patient’s driving pressures. When air is still flowing at the end of inspiration, a ventilator inspiratory pause will be required to read plateau pressure.

PEEP value is established by the ventilator for the first patient. Conversely, the second patient circuit has an adjustable in-line threshold valve (PEEP valve). The threshold value established with this valve in its expiratory circuit is the sum of the PEEP value shown in the monitor and the one set in the adjustable valve. The reading of the threshold value established by the position of the PEEP valve knob is not required. The ACRA manometers directly allow the user to read the values of PEEP, and the position of the knob can be adjusted from this reading.

The set of one-way valves prevent the flow from moving in unwanted directions. The one-way and threshold valves of the expiratory circuit should be selected to produce low values of pressure drop and seal adequately at low and high pressure differences.

A short circuit tubing between inspiratory and expiratory limbs, also proposed by [13], ensures compatibility of the system with different ventilator controllers.

As the ACRA system introduces pressure drops in the respiratory circuit, unknown to the ventilator controller, the feedback loop that enforces the different goal pressures throughout a respiration cycle might spiral out of control if the system never reaches its target. This is the case while measuring the target PIP from the expiration circuit, as the pressure drop introduced by the ACRA imposes an effective PIP which might be below target by a noticeable margin. Depending on the monitoring enforced, this could either raise an alarm, or simply lead to a feedback loop in the inlet regulation providing a higher flow rate and, eventually, delivering excessively high volumes to the patients. Also, a high discrepancy between inspiratory and expiratory pressures could lead to monitoring errors or false alarms [13].

Therefore, to establish an adequate protocol independent of both the ventilator considered and the feedback pressure probe location, it is beneficial to use short-circuit inspiratory and expiratory circuits as indicated in Fig 1(green line in single-line diagram) with a small size tubing (i.e. 6 mm in diameter). With this scheme, the ventilator feedback control system senses a single ‘virtual’ patient with a small resistance (mainly that of the short-circuit tubing) and a large compliance (mainly those of both patient lungs). Hence, the controller will target the parameters fixed on the monitor and adjust internal variables to obtain the predetermined goal of inspiration as a consequence of any disturbance with this patient configuration.

In [18] it has been emphasized the need to monitor the delivered tidal volume to each patient. The reading on the ventilator screen of the tidal volume with the ACRA corresponds to the value issued from the addition of volumes of both patients. To determine individual values, it is always possible to interpose in the inspiratory branch of a patient a flow sensor. Also, individual tidal volumes can be directly read on the ventilator screen by clamping the endotracheal tubing of one patient during a few cycles.

## Appendix B: Model parameters estimation

In our experimental set up, the values of lung resistance and compliance depend nonlinearly on the flow and volume respectively. This means that the compliance of both patients depends on the volume of each lung (*C*_*i*_ = *f*(*V*_*i*_)) and the resistance of both patients depends on the flow ($R_{i}=f\left(\dot{V_{i}}\right)$). These functions were incorporated into the model extracted from the SmartLung 2000 characteristic curves presented by the manufacturer in the datasheet.

**Fig 13:**
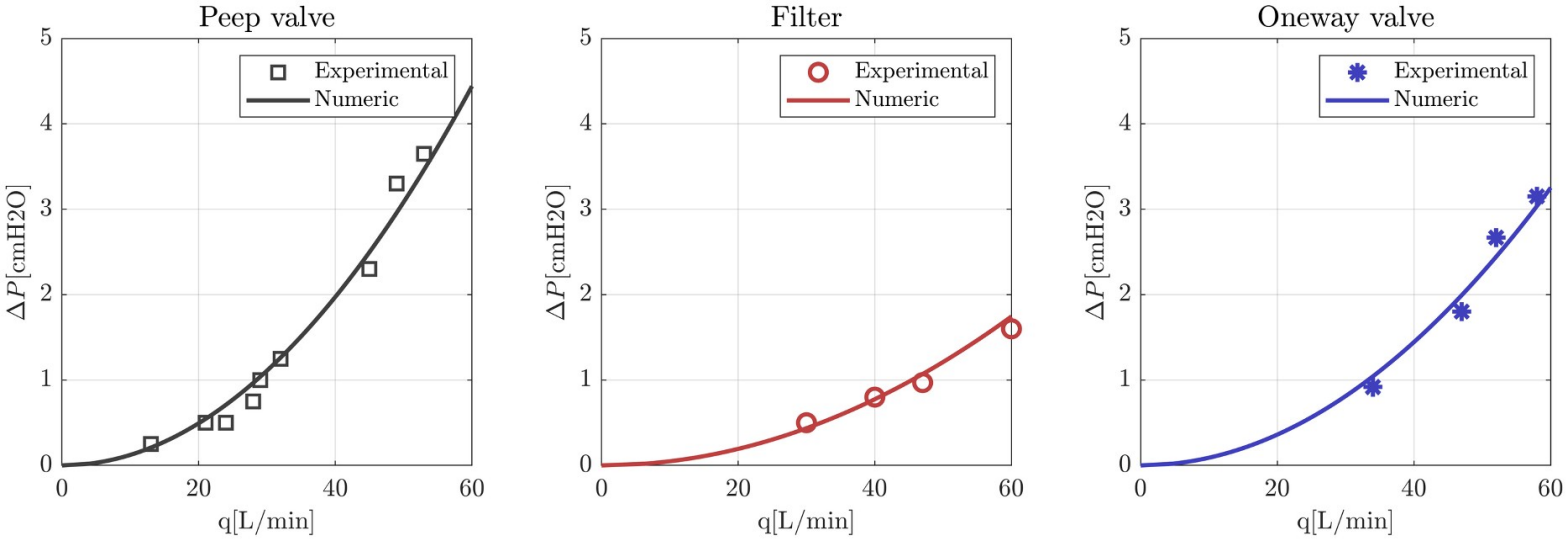
Characteristic curves of one-way valves, filters and PEEP valve.

A set of system parameters were obtained by fitting the results of laboratory experiments carried out for different flow conditions. The pressure loss curves of filters, one-way valves, and PEEP valve used in the model are shown together with the experimental measures in Fig 13, and the pressure loss coefficients are presented in Table 2.

**Table 2:** Pressure loss coefficients for circuit elements.

Element	Pressure loss coefficient [cm H_2_0 (L/min)^−2^]
PEEP Valve	1.24E-03
Filter	4.80E-04
One-way	9.01E-04

In all these fittings a quadratic dependence of pressure loss with flowrate was considered.

It is not easy to directly determine the expiratory valve characteristics of the ventilator. The governing law of this valve can take different complex forms and in many cases the geometry must be taken into account to correctly characterize it [26–28]. In our model, a linear pressure flow relation was considered ($\Delta P=R_{V}\dot{V}$). Constant *R*_*V*_ was determined through a minimization between pressure experimental measurements $P_{aw_{i}}^{exp}$ and the corresponding numerical output of the signal $P_{aw_{i}}^{num}\left(R_{V}\right)$. Experimental data was obtained for each patient circuit and for the expiratory circuit to ventilator. Flow rates and pressure signals were measured through orifice plates and differential pressure sensors connected to a FluxMed monitor. Numerical outputs were produced considering different values of (*R*_*V*_). To determine the more appropriate value of this parameter, we defined a functional *J*,

$$\begin{array}{c} J\left(R_{V}\right)={\left[\frac{P_{aw_{1}}^{exp}-P_{aw_{1}}^{num}\left(R_{V}\right)}{P_{aw_{1}}^{exp}}\right]}^{2}+{\left[\frac{P_{aw_{2}}^{exp}-P_{aw_{2}}^{num}\left(R_{V}\right)}{P_{aw_{2}}^{exp}}\right]}^{2}+{\left[\frac{P_{esp}^{exp}-P_{esp}^{num}\left(R_{V}\right)}{P_{esp}^{exp}}\right]}^{2} \end{array}$$ (4)

where $P_{aw_{1}}^{exp}$, $P_{aw_{2}}^{exp}$ and $P_{esp}^{exp}$ are patient 1 experimental airway pressure, patient 2 experimental airway pressure and experimental expiratory pressure respectively. Also, $P_{aw_{1}}^{num}$, $P_{aw_{2}}^{num}$ and $P_{esp}^{num}$ are patient 1 numerical airway pressure, patient 2 numerical airway pressure, and numerical expiratory pressure respectively all as a function of *R*_*V*_.

**Fig 14:**
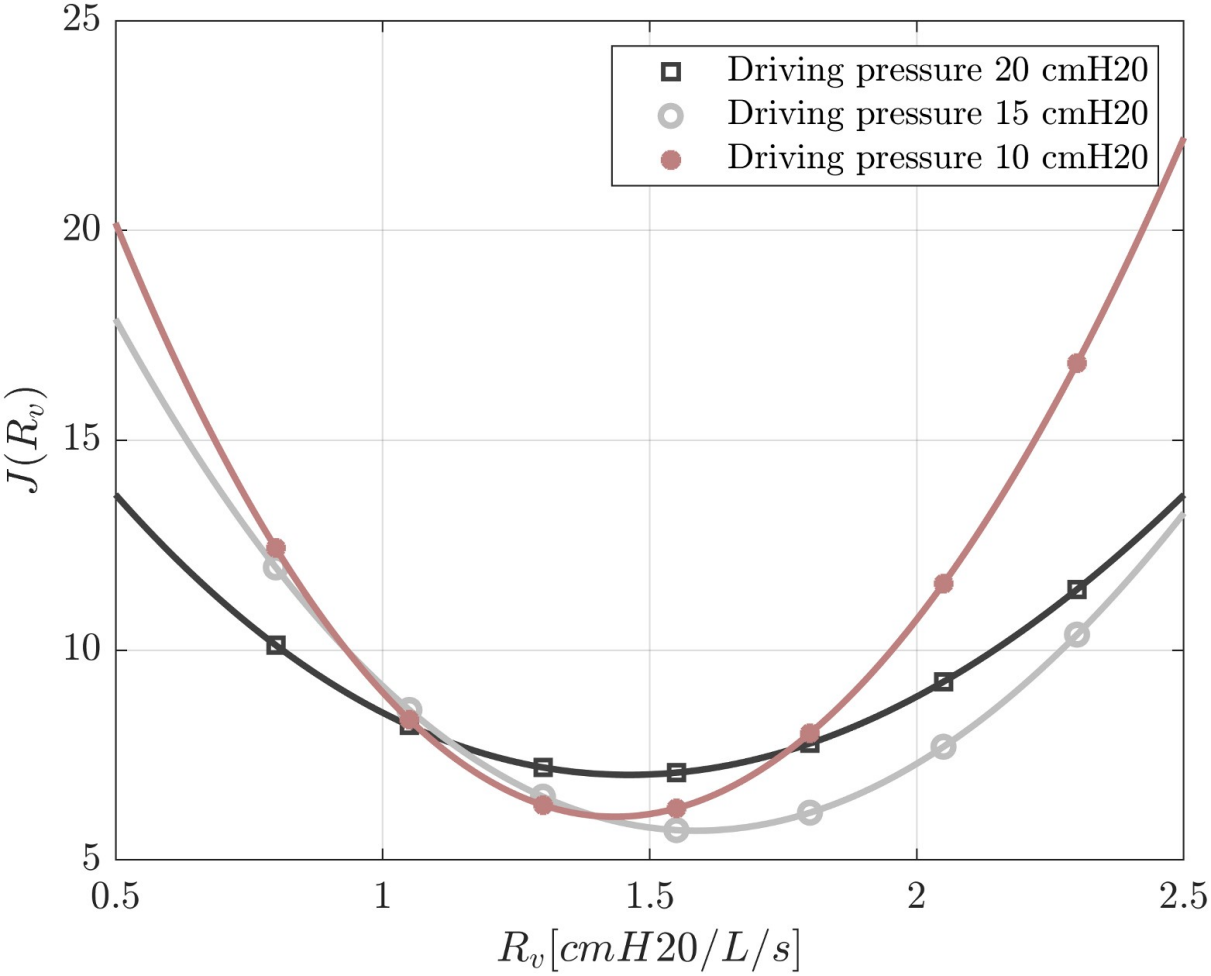
Estimation of ventilator resistance by minimizing functional J.

Functional *J* is plotted against *R*_*V*_ in Fig 14 for three driving pressures (Δ*P* ∈ [10, 15, 20] cm H_2_O. Therein, as *R*_*V*_ = 1.5 cm H_2_O (L/s)^−1^ minimizes *J*, this was taken as the exhalatory resistance value for all the tests.

## Appendix C: Simplified linear lumped element model

**Fig 15:**
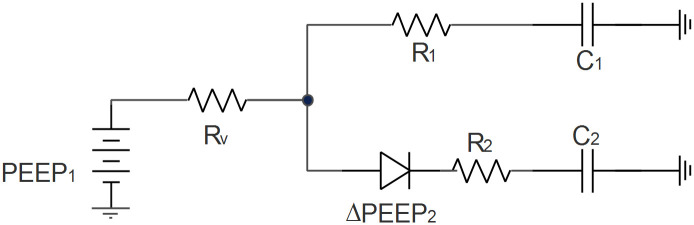
Linear lumped element model scheme of exhalation. The total charge in capacitors at time t = 0 corresponds to the air volume in each lung at the end of inspiration.

A schematic diagram of this simplified model is presented in Fig 15. Resistances *R*_1_ and *R*_2_ represent the airway resistance of each patient respectively. *R*_*V*_ represents the ventilator expiratory valve. Lung compliances are computed as *C*_1_ and *C*_2_ capacitance. Ventilator pressure is presented as a voltage source. The PEEP valve located on the patient 2 branch appears as a diode (resistance of this valve is considered in this analysis null in this analysis). Pressure losses generated on one-way valves, tube compliances, and tube resistances are neglected within this approach. In this simplified linear model, resistances and compliances are considered constant in time and independent of the involved flows or air volumes.

Kirchhoff circuit laws lead us to a system of differential equations presented in Eqs (5) and (6),

$$\begin{array}{c} \frac{V_{1}}{C_{1}}+R_{1}\dot{V_{1}}+R_{V}\left(\dot{V_{1}}+\dot{V_{2}}\right)={PEEP}_{1} \end{array}$$ (5)

$$\begin{array}{c} \frac{V_{2}}{C_{2}}+R_{2}\dot{V_{2}}+R_{V}\left(\dot{V_{1}}+\dot{V_{2}}\right)={PEEP}_{1}+\Delta PEEP_{2} \end{array}$$ (6)

where we identify flow rate of the patients with *V*_*i*_ (with *i* = 1, 2) and dots indicate time derivative. Denoting *PEEP*_2_ = Δ*PEEP*_2_ + *PEEP*_1_ and expressing the system of equation in a matrix form we get,

$$\begin{array}{c} \underset{\begin{array}{c} \bar{\bar{C}} \end{array}}{\left(\begin{array}{cc} \frac{1}{C_{1}} & 0\\ 0 & \frac{1}{C_{2}} \end{array}\right)}\underset{\begin{array}{c} \bar{V} \end{array}}{\left(\begin{array}{c} V_{1}\\ V_{2} \end{array}\right)}+\underset{\begin{array}{c} \bar{\bar{R}} \end{array}}{\left(\begin{array}{cc} R_{1}+R_{V} & R_{V}\\ R_{V} & R_{2}+R_{V} \end{array}\right)}\underset{\begin{array}{c} \dot{\bar{V}} \end{array}}{\left(\begin{array}{c} \dot{V_{1}}\\ \dot{V_{2}} \end{array}\right)}=\underset{\begin{array}{c} \bar{PEEP} \end{array}}{\left(\begin{array}{c} {PEEP}_{1}\\ {PEEP}_{2} \end{array}\right)} \end{array}$$ (7)

This equation system $\bar{\bar{C}}.\bar{V}+\bar{\bar{R}}.\dot{\bar{V}}=\bar{PEEP}$ is a system of first order linear nonhomogeneous differential equations. Therefore, the general solution of the problem will be the sum of the homogeneous and a particular solution.

This solution takes the form,

$$\begin{array}{c} \left(\begin{array}{c} V_{1}\left(t\right)\\ V_{2}\left(t\right) \end{array})=\alpha \bar{A_{1}}e^{-\frac{1}{|\lambda_{1}|}t}+\beta \bar{A_{2}}e^{-\frac{1}{|\lambda_{2}|}t}+(\begin{array}{c} C_{1}{PEEP}_{1}\\ C_{2}{PEEP}_{2} \end{array}\right) \end{array}$$ (8)

where the scalar λ_*i*_ and vector $\bar{A_{i}}$ are the eigenvalues and eigenvectors of matrix (${\bar{\bar{C}}}^{-1}\cdot \bar{\bar{R}}$) respectively. The eigenvalues λ_*i*_ can be computed as $\lambda_{i}=\frac{1}{2}(b\pm \sqrt{b^{2}-4c}$), where *b* = *C*_1_(*R*_1_ + *R*_*V*_) + *C*_2_(*R*_2_ + *R*_*V*_) and $c=C_{1}C_{2}\left(R_{1}+R_{V}\right)\left(R_{2}+R_{V}\right)-C_{1}C_{2}R_{V}^{2}$. The general expression for the eigenvectors can be seen on https://github.com/ACRA2020/Analytical-Solution. Finally, scalars *α* and *β* depend on the values of tidal volumes at the end of inspiration.

In order to obtain a tractable analytical solution, the assumption of null flow at the end of inspiration is made. Then, considering pressures at the end of inspiration *P*_*i*_ as the initial conditions of expiration, the constants *α* and *β* can be determined. It is worth mentioning, that in this case, the exhalatory driving pressure is equal to the driving pressure. For a more general case, the exhalatory driving pressure *DP*_*i*_ for each patient is the difference $DP_{i}=P_{i}-{PEEP}_{i}$ (with *P*_*i*_ the pressure of patient i at the initiation of exhalation) and,

$$\begin{array}{c} (\begin{array}{c} DP_{1}C_{1}\\ DP_{2}C_{2} \end{array})=\alpha \bar{A_{1}}+\beta \bar{A_{2}} \end{array}$$ (9)

Note that this maximum pressure difference is, in this case, intimately related with the driving pressure defined as the plateau airway pressure minus PEEP.

These last equations indicate that constants *α* and *β* depend on the driving pressures and compliances of each patient. Therefore, this analysis shows that the speed at which lungs go from an initial state (that is, function of the pressure at the end of inspiration) to the final state depends on *R*_1_, *R*_2_, *R*_*V*_, *C*_1_, *C*_2_, *DP*_1_ and, *DP*_2_.

With the aim of identifying the relevant physical quantities that dictate the exhalatory fluid dynamics, we nondimensionalize the simplified model equations with patient 1 parameters.

We define the nondimensional volume as, *v*_1_ = *V*_1_/(*C*_1_ *DP*_1_) and *v*_2_ = *V*_2_/(*C*_1_ *DP*_1_) and the nondimensional flow as, ${\dot{v}}_{1}=$ $\dot{V_{1}}\left(R_{1}/DP_{1}\right)$ and ${\dot{v}}_{2}=$ $\dot{V_{2}}\left(R_{1}/DP_{1}\right)$. Taking into account these definitions, the homogeneous system of Eqs (5) and (6) can be written in a new and equivalent system of first order nondimensional linear homogeneous differential equations as:

$$\begin{array}{c} v_{1}+\dot{v_{1}}+\frac{R_{V}}{R_{1}}\left(\dot{v_{1}}+\dot{v_{2}}\right)=0 \end{array}$$ (10)

$$\begin{array}{c} \frac{C_{1}}{C_{2}}v_{2}+\frac{R_{2}}{R_{1}}\dot{v_{2}}+\frac{R_{V}}{R_{1}}\left(\dot{v_{1}}+\dot{v_{2}}\right)=0 \end{array}$$ (11)

By simple inspection of Eqs (10) and (11), the homogeneous system of equations presented is governed by three groups of parametric relations, *R*_2_/*R*_1_, *C*_2_/*C*_1_, and *R*_*V*_/*R*_1_ The same reasoning can be applied for Eq (9). Then, it can be concluded that the general solution of Eqs (5) and (6) depend on the following ratios, *R*_2_/*R*_1_, *C*_2_/*C*_1_, *R*_*V*_/*R*_1_, *DP*_2_/*DP*_1_, *PEEP*_2_/*PEEP*_1_.

The use of patient 1 parameters to undertake the nondimensionalization of the equations establishes that the expiratory characteristic times obtained are referred to the time constant *R*_1_ *C*_1_. However, to facilitate the analysis without loosing generality, we preferred in the manuscript to present the expiratory characteristic times referred to the available exhalatory time *t*_*ex*_.

## References

[1] Darowski Marek and Englisz Marek. Artificial ventilation of the lungs for emergencies. *Frontiers of medical and biological engineering*, 10:177–183, 2000. 10.1163/15685570052062576 11014679

[2] Shriya Srinivasan, Khalil B Ramadi, Francesco Vicario, Declan Gwynne, Alison Hayward, Robert Langer, et al. Individualized system for augmenting ventilator efficacy (isave): A rapidly deployable system to expand ventilator capacity. *[Preprint] bioRxiv*, 2020. 10.1126/scitranslmed.abb9401 PMC7259824 32424018

[3] Kheyfets Vitaly, Lammers Steven, Wagner Jennifer, Bartels Karsten, and Smith Bradford. PEEP/FIO_2_ ARDSNet scale grouping of a single ventilator for two patients: Modelling tidal volume response. *Respiratory Care*, 65:1094–1103, 2020. 10.4187/respcare.07931 32712582 PMC7538006

[4] Branson Richard and Rubinson Lewis. A single ventilator for multiple simulated patients to meet disaster surge. *Academic emergency medicine*: *official journal of the Society for Academic Emergency Medicine*, 13:1352–1353, 2007. 10.1197/j.aem.2006.10.002 17158729

[5] Branson Richard, Blakeman Thomas, Robinson Bryce, and Johannigman Jay. Use of a single ventilator to support 4 patients: Laboratory evaluation of a limited concept. *Respiratory Care*, 57:399–403, 2012. 10.4187/respcare.01236 22005780

[6] Pearson Steven, Hall Jesse, and Parker William. Two for one with split-or co-ventilation at the peak of the covid-19 tsunami: Is there any role for communal care when the resources for personalised medicine are exhausted? *Thorax*, 75:444–445, 2020. 10.1136/thoraxjnl-2020-214929 32327565 PMC7675921

[7] Single ventilator use to support multiple patients. *ECRI*, *Clinical Evidence Assessment*, 2020.

[8] Neyman Greg and Irvin Charlene Babcock. A single ventilator for multiple simulated patients to meet disaster surge. *Academic emergency medicine*, 13:1246–1249, 2006. 10.1197/j.aem.2006.05.009 16885402 PMC7164837

[9] Herrmann Jacob, Cruz Andrea, Hawley Monica, Branson Richard, and Kaczka David. Shared ventilation in the era of covid-19: A theoretical consideration of the dangers and potential solutions. *Respiratory Care*, 65:932–945, 2020. 10.4187/respcare.07919 32376612

[10] Clarke A. L., Stephens A. F., S. Liao, Byrne T. J., and Gregory S. D. Coping with covid-19: ventilator splitting with differential driving pressures using standard hospital equipment. *Anaesthesia*, 75:872–880, 2020. 10.1111/anae.15078 32271942 PMC7262199

[11] Han Jay S, Mashari Azad, Singh Devin, Dianti Jose, Goligher Ewan, Long Michael, et al. Personalized ventilation to multiple patients using a single ventilator: Description and proof of concept. *Critical Care Explorations*, 2:e0118, 2020. 10.1097/CCE.0000000000000118 32671348 PMC7259561

[12] Solís-Lemus José A., Costar Edward, Doorly Denis, Kerrigan Eric C., Kennedy Caroline H., Tait Frances, et al. A simulated single ventilator/dual patient ventilation strategy for acute respiratory distress syndrome during the covid-19 pandemic. *Royal Society Open Science*, 7:200585, 2020. 10.1098/rsos.200585 32968521 PMC7481711

[13] Micha Sam Brickman Raredon, Clark Fisher, Paul Heerdt, Ranjit Deshpande, Steven Nivison, Elaine Fajardo, et al. Pressure-regulated ventilator splitting (prevents): A COVID-19 response paradigm from YALE university. *[Preprint] medRxiv*, 2020.

[14] Paladino Lorenzo, Silverberg Mark, Charchaflieh Jean, Eason Julie, Wright Brian, Palamidessi Nicholas, et al. Increasing ventilator surge capacity in disasters: Ventilation of four adult-human-sized sheep on a single ventilator with a modified circuit. *Resuscitation*, 77:121–126, 2008. 10.1016/j.resuscitation.2007.10.016 18164798

[15] Smith R and black J.M. Simultaneous ventilation of two healthy subjects with a single ventilator. *Resuscitation*, 80:1087, 2009. 10.1016/j.resuscitation.2009.05.018 19573974 PMC9912358

[16] Levin Matthew A., Shah Anjan, Shah Ronak, Kane Erica, Zhou George, Eisenkraft James B., et al. For the Mount Sinai HELPS Innovate Group; Differential Ventilation Using Flow Control Valves as a Potential Bridge to Full Ventilatory Support during the COVID-19 Crisis. *Anesthesiology*, 892:904, 2020. 10.1097/ALN.0000000000003473 PMC7359901 32639236

[17] Andrew Plummer, Jonathan Du Bois, Siu Lee, Patrick Magee, Jens Roesner, and Harinderjit Gill. The bath RC model: a method to estimate flow restrictor size for dual ventilation of dissimilar patients. *[Preprint] MedRxiv*, 2020. 10.1371/journal.pone.0242123 PMC7668571 33196687

[18] Chatburn Robert, Branson Richard, and Umur Hatipoğlu. Multiplex ventilation: A simulation-based study of ventilating two patients with one ventilator. *Respiratory Care*, 65:920–931, 2020. 10.4187/respcare.07882 32345741

[19] Marini John, Crooke Philip, and Truwit Jonathon. Determinants and limits of pressure-preset ventilation: A mathematical model of pressure control. *Journal of applied physiology (Bethesda*, *Md*.:*1985)*, 67:1081–1092, 1989. 10.1152/jappl.1989.67.3.1081 2676950

[20] Verheecke G and Gilbertson Alfred. Compliance of the tubing compartment of lung ventilators. *Intensive care medicine*, 7:309–310, 1981. 10.1007/BF01709728 6948881

[21] Mínimos clínicamente aceptables para el desarrollo de dispositivos capaces de dar soporte ventilatorio durante la pandemia generada por covid 19. *Sociedad Argentina de Terapia Intensiva*, 2020.

[22] Brusasco Vito, Beck Kenneth, Crawford Michelle, and Rehder K. Resonant amplification of delivered volume during high-frequency ventilation. *Journal of applied physiology*, 60:885–892, 1986. 10.1152/jappl.1986.60.3.885 2937763

[23] WB Van de Graaff and MJ Tobin. Monitoring of lung mechanics and work of breathing. *Principles and practice of mechanical ventilation*, pages 967–1003, 1994.

[24] Venegas Jose, Harris Robert, and Simon Brett. A comprehensive equation for the pulmonary pressure-volume curve. *Journal of applied physiology*, 84:389–395, 1998. 10.1152/jappl.1998.84.1.389 9451661

[25] Jiao Guang-Yu and Newhart John W. Bench study on active exhalation valve performance. *Respiratory Care*, 53:1697–1702, 2008. 19025705

[26] Banner M, Lampotang Samsun, Boysen Philip, Hurd T, and Desautels D. Flow resistance of expiratory positive-pressure valve systems. *Chest*, 90:212–217, 1986. 10.1378/chest.90.2.212 3525024

[27] Kayaleh R and Wilson A. Mechanisms of expiratory valves resistance. *Am Rev Respir Dis*, 137:1390–1394, 1988. 10.1164/ajrccm/137.6.1390 3202376

[28] Dick W, Milewski L., Lotz P, and Ahnefeld F.W. A new positive end expiratory pressure valve for manually operated artificial ventilation. *Resuscitation*, 6:1–8, 1978. 10.1016/0300-9572(78)90030-8 353930

